# Is glycaemic control associated with dietary patterns independent of weight change in people newly diagnosed with type 2 diabetes? Prospective analysis of the Early-ACTivity-In-Diabetes trial

**DOI:** 10.1186/s12916-022-02358-5

**Published:** 2022-04-18

**Authors:** James Garbutt, C. England, A. G. Jones, R. C. Andrews, R. Salway, L. Johnson

**Affiliations:** 1 Centre for Exercise, Nutrition and Health Sciences, School for Policy Studies, University of Bristol, 8 Priory Road, Bristol, BS8 1TZ UK; 2 NIHR Bristol Biomedical Research Centre, University Hospitals Bristol NHS Foundation Trust and University of Bristol, Bristol, UK; 3 Institute of Biomedical and Clinical Science, University of Exeter Medical School, University of Exeter, Exeter, UK; 4 Diabetes and Endocrinology, Royal Devon and Exeter Hospital, Exeter, UK

**Keywords:** HbA1c, Type 2 diabetes, Diet, Dietary patterns, Reduced rank regression, Carbohydrates, Fat, Fibre, Energy-density, Weight loss

## Abstract

**Background:**

It is unclear whether diet affects glycaemic control in type 2 diabetes (T2D), over and above its effects on bodyweight. We aimed to assess whether changes in dietary patterns altered glycaemic control independently of effects on bodyweight in newly diagnosed T2D.

**Methods:**

We used data from 4-day food diaries, HbA1c and potential confounders in participants of the Early-ACTivity-In-Diabetes trial measured at 0, 6 and 12 months. At baseline, a ‘carb/fat balance’ dietary pattern and an ‘obesogenic’ dietary pattern were derived using reduced-rank regression, based on hypothesised nutrient-mediated mechanisms linking dietary intake to glycaemia directly or via obesity. Relationships between 0 and 6 month change in dietary pattern scores and baseline-adjusted HbA1c at 6 months (*n* = 242; primary outcome) were assessed using multivariable linear regression. Models were repeated for periods 6–12 months and 0–12 months (*n* = 194 and *n* = 214 respectively; secondary outcomes).

**Results:**

Reductions over 0–6 months were observed in mean bodyweight (− 2.3 (95% CI: − 2.7, − 1.8) kg), body mass index (− 0.8 (− 0.9, − 0.6) kg/m^2^), energy intake (− 788 (− 953, − 624) kJ/day), and HbA1c (− 1.6 (− 2.6, -0.6) mmol/mol). Weight loss strongly associated with lower HbA1c at 0–6 months (*β* = − 0.70 [95% CI − 0.95, − 0.45] mmol/mol/kg lost). Average fat and carbohydrate intakes changed to be more in-line with UK healthy eating guidelines between 0 and 6 months. Dietary patterns shifting carbohydrate intakes higher and fat intakes lower were characterised by greater consumption of fresh fruit, low-fat milk and boiled/baked potatoes and eating less of higher-fat processed meats, butter/animal fats and red meat. Increases in standardised ‘carb/fat balance’ dietary pattern score associated with improvements in HbA1c at 6 months independent of weight loss (*β* = − 1.54 [− 2.96, − 0.13] mmol/mol/SD). No evidence of association with HbA1c was found for this dietary pattern at other time-periods. Decreases in ‘obesogenic’ dietary pattern score were associated with weight loss (*β* = − 0.77 [− 1.31, − 0.23] kg/SD) but not independently with HbA1c during any period.

**Conclusions:**

Promoting weight loss should remain the primary nutritional strategy for improving glycaemic control in early T2D. However, improving dietary patterns to bring carbohydrate and fat intakes closer to UK guidelines may provide small, additional improvements in glycaemic control.

**Trial registration:**

ISRCTN92162869. Retrospectively registered on 25 July 2005

**Supplementary Information:**

The online version contains supplementary material available at 10.1186/s12916-022-02358-5.

## Background

Diet is one of the cornerstones of treatment for patients diagnosed with type 2 diabetes [1, 2]. Nutritional guidelines for glycaemic management focus on individualised recommendations rather than specifying a ‘one-size-fits-all’ approach [1, 2]. Improved HbA1c concentrations are consistently associated with weight-loss through caloric deficit achieved by a range of specific dietary changes [3, 4]. However, it remains unclear if specific dietary patterns can offer further benefits to glycaemic control beyond their effect on bodyweight.

Evidence for appropriate dietary intakes in type 2 diabetes, notably relating to carbohydrates, is inconsistent [5, 6]. Separating potential effects of dietary composition change from the known effects of weight change on glycaemic control is difficult as both typically co-occur. Dietary trials in type 2 diabetes have also tended to focus on manipulating intakes of only single nutrients, such as comparing ‘low-carb’ vs ‘high-carb’ diets, but this bears little resemblance to real-world eating behaviours. People eat foods comprising of multiple nutrients. Changing food intakes to modify one nutrient inevitably changes many nutrient intakes simultaneously. Capturing these multiple dietary changes in the form of a ‘dietary pattern’, alongside an understanding of the mechanisms through which these might act, is key for appreciating any specific effects of diet in type 2 diabetes.

**Fig. 1:**
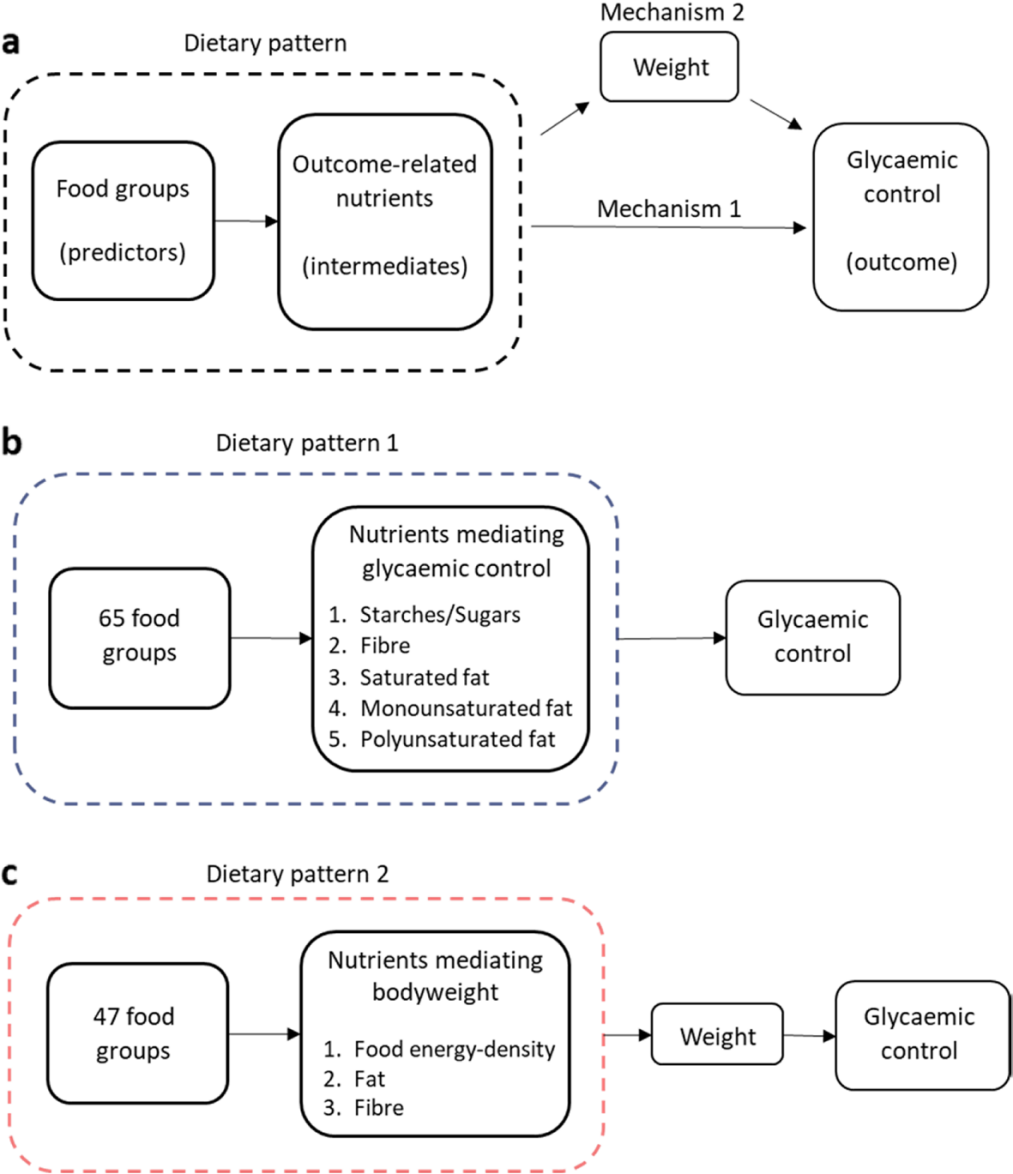
Changes in dietary patterns constructed to explain differences in intakes of multiple nutrients simultaneously are explored for independent (mechanism 1) and weight-dependent (mechanism 2) associations with changes in glycaemic control. **a** General pathway diagram. **b** Mechanism 1—dietary pattern 1 hypothesised to directly associate with glycaemic control. **c** Mechanism 2—dietary pattern 2 hypothesised to indirectly associate with glycaemic control via effects on bodyweight

Reduced-rank regression (RRR) is an analytical technique that identifies dietary patterns in reported food intakes by combining existing knowledge and theory of diet-disease mechanisms with data-driven analysis of real-world eating behaviours [7]. RRR can establish the relative importance of different nutrient-mediated mechanisms potentially linking diet directly to glycaemic control or indirectly via weight loss (see Fig. 1a–c). Identifying key foods that explain the most variation in these specific diet-disease mechanisms may in turn offer high-priority food targets for dietetic management of type 2 diabetes, therefore offering significant clinical potential.

To our knowledge, no study has applied RRR to identify dietary patterns associated with glycaemic control in patients with type 2 diabetes. However, RRR has been applied to derive dietary patterns associated with type 2 diabetes incidence [7–12]. Reduced incidence has generally been associated with dietary patterns high in wholegrains and vegetables and low in refined-grains, sugar-sweetened beverages and processed meats. In the majority of these studies, inflammatory biomarkers were used as the intermediate mechanisms linking food intake with type 2 diabetes, rather than nutrients. Inflammatory biomarkers are subject to multiple influences beyond dietary patterns and therefore may not capture the dietary variation most relevant to type 2 diabetes compared with using nutrient intermediates [13]. Associations between biomarker-mediated dietary patterns and type 2 diabetes incidence are also potentially self-fulfilling, as the intermediate biomarkers used in the dietary pattern’s construction associate with incidence a priori. Instead, nutrients as potential diet-disease intermediates will be more proximal to dietary intakes, and should only produce self-fulfilling associations in as far as the chosen nutrients are true mediators between diet and disease.

The nutritional changes hypothesised to optimise glycaemic control may differ from the nutritional changes required to optimise weight loss. For instance, several nutrients potentially directly link diet with glycaemic control (‘mechanism 1’); fibre moderates post-prandial blood glucose excursions caused by starchy and sugary carbohydrate consumption, whilst replacing saturated with unsaturated fats has been observed to benefit insulin sensitivity [14–16]. Several other nutrient factors have the potential to link diet to glycaemic control via their effects on bodyweight (‘mechanism 2’), such as dietary energy-density and chief calorie contributors and appetite buffers like total fat and fibre [17, 18] (Fig. 1a–c). Understanding the effects of simultaneous changes in these nutrients in the form of a dietary pattern, as derived using RRR, allows exploration of these nutrients’ combined importance in their potential impact on glycaemic control.

In this study, we investigate whether changes in dietary patterns that explore nutrient-mediated disease mechanisms were independently associated with changes in HbA1c observed over 6 or 12 months of the Early-ACTivity-In-Diabetes (Early-ACTID) trial [ISRCTN Registry: 92162869] [19].

## Methods

### Sample

Data came from the Early-ACTID trial [19, 20], a 12-month, multi-centre, parallel-group randomised controlled trial involving 593 adults diagnosed in the previous 5–8 months with type 2 diabetes. Recruitment took place from December 2005 to September 2008 within South-West England. Participants were randomised to either a usual care, dietary intervention or diet and physical activity intervention group. During the first 6 months of the study, glucose-lowering medications were not changed. Trial endpoints were HbA1c and blood pressure at 6 months (primary) and 12 months (secondary) post-intervention. The study was approved by the Bath Research Ethics Committee (05/Q2001/5), and all participants provided written informed consent.

### Intervention

Usual care consisted of standard dietary and exercise advice at 0 and 12 months, with an interim review by study doctors and nurses at 6 months, where no further advice was given. The diet intervention aimed to enable and maintain 5–10% weight loss through a non-prescriptive dietary intervention based on 2003 Diabetes UK nutrition guidelines [21] and UK Food Standards Agency’s ‘Balance of Good Health’ [22]. Specifically, participants were encouraged to base meals on starchy carbohydrates and choose higher-fibre/wholegrain options, reduce added sugars, increase oily fish and reduce fatty and processed meat intakes and choose lower-GI and energy-density foods. Guidance also included maintaining a regular meal pattern alongside general portion-size control. Dietitians met with participants at randomisation and every 3–4 months, with study nurses reinforcing advice every 6-weeks. The diet and physical activity intervention consisted of the same dietary intervention as the diet-only group. Participants were however advised to do an additional ≥ 30 min walk on ≥ 5 days per week. Study nurses also discussed physical activity during the 6-weekly appointments. Total contact time was the same in both intervention groups.

### Dietary data

Diet was self-reported using 4-day food diaries covering two weekdays and two weekend days. All foods and drinks (including alcohol) were reported with estimated portion sizes using household measures or package weights, noting brands and cooking methods where appropriate. Food diaries were coded according to the University of Bristol’s Centre for Exercise, Nutrition and Health Sciences food diary codebook. This codebook is based on the INTERMAP study [23], common foods in the first 6 years (2008–2014) of the UK National Diet and Nutrition Survey (NDNS) [24], portion sizes from the 2006 *Final Technical Report to the Food Standards Agency on Typical Food Portion Sizes in Adults* [25], and coding rules taken from the UK ALSPAC [26] and AIRWAVE [27] study codebooks.

Food diaries were coded by two researchers and quality-checked by two others in line with best practise for minimising coding errors [23]. The *Diet In Data Out* nutritional analysis software [28] was used for analysing 0- and 6-month diaries, and *DietPlan* (v7; Forestfield Software Limited, UK) was used for 12-month diaries. Diet analyses used nutrient data published in the 2002 UK *Composition of Foods Integrated Dataset* (COFID) [29] to more closely match food composition at the time of the trial, or if missing, the updated 2015 COFID database [30].

### Diet pattern derivation

Average daily percentage total energy intake (TEI) were calculated using updated Atwater factors [31] for starches and sugars combined, saturated fats (SFA), monounsaturated fats (MUFA), polyunsaturated fats (PUFA) and total fat intakes using: 100*energy from nutrient (kJ)/total energy (kJ). Average daily fibre-density was calculated using total fibre (g)/total energy (MJ). Average daily dietary energy-density (DED) was calculated using total food energy (kJ)/total food weight (g), excluding drinks to prevent inappropriately diluting estimates [32].

All dietary patterns were derived using RRR. Fibre-density (g/MJ) and percentage energy from starches and sugars, SFA, MUFA and PUFA (%TEI) were used as intermediate variables for deriving a dietary pattern based on evidence that individual macronutrients directly affect glycaemia [14–16] (mechanism 1; Fig. 1b). DED (kJ/g), total fat (%TEI) and fibre-density (g/MJ) were used as intermediate variables for deriving a dietary pattern hypothesised to indirectly associate glycaemic control via bodyweight (mechanism 2; Fig. 1c), replicating previous methods [17, 18, 33, 34]. For dietary pattern mechanism 1, food items were allocated to 65 groups based on culinary usage and to maximise differences in fat and carbohydrate quality (Additional file 1: Table S1). For dietary pattern mechanism 2, 47 food groupings based on previous studies were used [18] (Additional file 1: Table S1). Average intake of each food group was calculated in g/day at 0, 6 and 12 months for each participant.

RRR derives a dietary pattern score for each participant computed from their individual standardised food group intakes weighted by dietary pattern loadings derived at group-level. Pattern scores are increased when participants report eating more of food groups with higher (positive) pattern loadings or eating less of food groups with lower (negative) pattern loadings. RRR produces as many dietary patterns as intermediate variables used; hence, for dietary pattern mechanism 1 this was 5, and for dietary pattern mechanism 2, this was 3 patterns. To identify a single score for each pattern that captured the combination of food groups explaining most variation in specified nutrient intermediates, we only retained the first patterns for subsequent analyses. To confirm whether the pattern structures (i.e. the size or direction of food group loadings for pattern scores) changed over time, we repeated the RRR independently at 6 and 12 months and compared the first patterns derived at these timepoints with the first patterns at baseline using Tucker’s congruence coefficient (CC) [35]. After confirming patterns were similar (CC > 0.85), food group pattern loadings at baseline were used to compute dietary pattern scores at 6 and 12 months, thus allowing changes in adherence to the same dietary pattern structure to be measured. We also assessed congruence between dietary pattern 2 and dietary patterns derived using identical methods in the UK NDNS [18], to assess stability of this pattern between populations.

### Misreporting of energy intake

Dietary misreporting at baseline was assessed via an individualised method [36] using a ratio of reported energy intake to estimated energy requirement, calculated from standard equations [37] (Additional file 1: Supplementary information S1 [38, 39]). Assuming energy balance, energy intake is expected to be equal to estimated energy requirements. Early-ACTID was a weight-loss trial, so whilst energy balance may be assumed at baseline, it is an unreasonable assumption during the intervention. Therefore, baseline misreporting status was used to assign misreporting status at later timepoints, as misreporting has previously been seen to track within individuals [40]. As few over-reporters were identified (*n* = 4), these were combined with plausible-reporters and a binary categorical variable (under-reporter and plausible-reporter) was used in analyses.

### Covariates

Diet, physical activity, anthropometry, medications, clinical and haematological measures including HbA1c were assessed at three timepoints (0, 6 and 12 months post-randomisation). HbA1c was measured in plasma using HPLC in a single laboratory. Oral hypoglycaemic agents (OHAs), namely metformin, sulphonylureas and glitazones, were recorded by trial clinicians as type and dose. Physical activity was assessed over 7 days via waist-worn, uni-axial accelerometers (*Actigraph GT1M; Actigraph LLC, Pensacola, FL, USA*), with data processing as detailed previously [41]. Participants were additionally scored against the 2007 UK Index of Multiple Deprivation (IMD) based on their home postcode at baseline [42]. Covariates used for analyses were 0-, 6-, and 12-month percentages of maximum OHA medication dose, average daily total physical activity, bodyweight and TEI, and baseline age, sex and dietary-misreporting status.

### Statistical analysis

Variables were described with the use of mean (standard deviation (SD) or 95% confidence interval (95%CI)) if normally distributed or median (quartile 1, quartile 3) otherwise. Associations between participant characteristics and changes in dietary patterns and high pattern loading food groups were explored by describing the sample by quintile of dietary pattern score change. To help understand what a 1-SD change in dietary pattern score means, nutrient intake changes relating to a 1-SD increase in dietary pattern score were calculated using simple linear regression, with dietary pattern score as predictor and either DED, fibre-density or percentage energy from the relevant nutrient as outcomes.

The primary outcome of this study was change in HbA1c over 0–6 months (0–6 m), a period when no changes in medications were made and thus diet had the most potential to affect HbA1c. Changes in HbA1c during 6–12 months (6–12 m) or 0–12 months (0–12 m) were explored as secondary outcomes, adjusting for changes in medications during the latter half of the trial. Trial periods were thus modelled separately to distinguish effects attributable to lifestyle only to that of lifestyle and medications combined.

A series of multivariable linear regressions were used to assess whether dietary pattern changes during 0–6 m were associated with glycaemic control, as measured through change in HbA1c. *Model 1* estimated the unadjusted association between each dietary pattern score change (exposure) and HbA1c change using end-of-period (6-month) HbA1c as the outcome, adjusting for start-of-period (baseline) HbA1c and dietary pattern score. *Model 2* estimated the association independent of potential confounders by adding to model 1, age, sex, misreporting status and period-change in total physical activity. Model 3 estimated potential mediation by adding period-change in TEI and bodyweight to model 2. To assess the subsequent 6-month and longer-term association between dietary pattern change and HbA1c, we repeated models 1–3 for 6–12 m and 0–12 m periods. In these models, we additionally adjusted for OHA medication change within models 2 and 3. Units of dietary pattern change effect estimates within these models were for an equivalent 1-SD change in baseline dietary pattern score. We considered *p* < 0.05 being evidence of association.

### Sensitivity analyses

We ran a series of sensitivity analyses to check our assumptions relating to missing data, linearity of associations, interactions by sex, model adjustment with trial arm and associations between bodyweight and HbA1c change (Additional file 1: Supplementary information S2).

Analyses were performed in *Stata* (v15; StataCorp LLC, College Station, TX, USA), with the RRR procedure incorporating *SAS* (v9; SAS Institute, NC, USA) (Additional file 1: Supplementary information S3).

## Results

### Sample characteristics

**Fig. 2:**
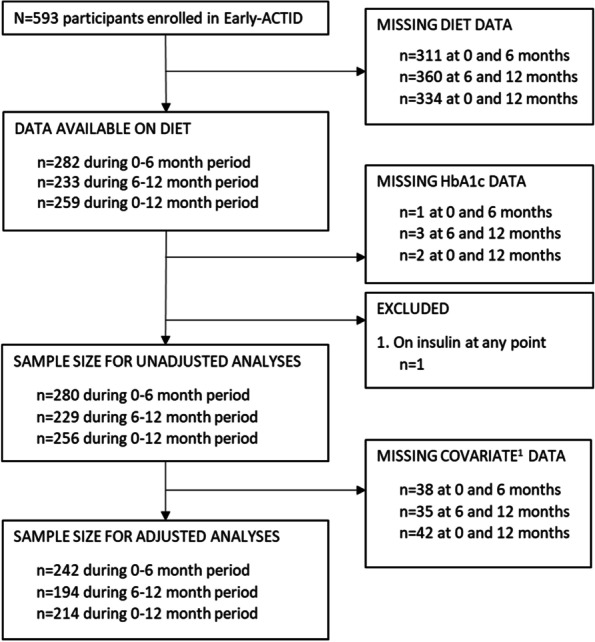
Sample size flow chart. ^1^ Covariates were age, sex, bodyweight, energy intake, total physical activity, under-reporting status and metformin, sulphonylurea and glitazone dose

Complete data for our primary analysis between 0 and 6 m was available in *n* = 242 participants (41% of those enrolled at baseline) (Fig. 2), of which 67% were male with median age 62 years, weight 86.5 kg, body mass index (BMI) 29.5 kg/m^2^ and HbA1c 47 mmol/mol (6.5%) (Tables 1 and 2). Baseline characteristics of all trial participants (*n* = 593) and of those included in our secondary analyses (*n* = 194 at 6-12 m; *n* = 214 at 0–12 m) are shown in Additional file 1: Table S2. Fewer participants in the secondary analyses came from the usual care group (7% vs. 17%) and were more likely to be male (70–71%) and slightly older (median baseline age 63 years), but were otherwise similar to participants in the 0-6 m analyses.

**Table 1:** Baseline and 0–6-month change characteristics for *n* = 242 participants with complete data during 0–6 months

		0–6 m participant characteristics (models 1a-3)
***n*** (% full cohort)		242 (41%)
**Arm**, *n* (% sample)		
	Usual care	16 (7%)
	Diet	115 (48%)
	Diet and exercise	111 (46%)
**Male**, *n* (%)		163 (67%)
**White ethnicity**, *n* (%)		236 (98%)
**Smoker at baseline**, *n* (%)		17 (7%)
**Age**, years		62 (57, 69)
**Time since diagnosis**, years		0.5 (0.4, 0.6)
**IMD score**		12.6 (6.4, 18.9)
**Total activity**, counts/min		291 (226, 363)
**Total activity change 0–6 m**, counts/min		16 (− 44, 91)
**MVPA**, mins/day		21 (13, 36)
**MVPA change 0–6 m**, mins/day		3 (− 6, 18)
**Weight**, kg		86.5 (77.1, 94.0)
**Weight change 0–6 m**, kg		− 2.1 (− 3.9, − 0.1)
**BMI**, kg/m^2^		29.5 (27.3, 32.7)
**BMI change 0–6 m**, kg/m^2^		− 0.7 (− 1.4, 0.0)
**HbA1c**, mmol/mol		47 (43, 54)
**HbA1c**, %		6.5 (6.1, 7.1)
**HbA1c change 0–6 m**, mmol/mol		− 2.2 (− 5.5, 3.3)
**HbA1c change 0–6 m**, %		− 0.2 (− 0.5, 0.3)
**OHA prescription**, *n* (%)		
	Metformin	85 (35%)
	Sulphonylurea	22 (9%)
	Glitazone	2 (1%)
**Baseline under-reporters**, *n* (%)		142 (56%)

Data presented as median (quartile 1, quartile 3) or *n* (%). *IMD* index of multiple deprivation, *MVPA* moderate-vigorous physical activity, *OHA* oral-hypoglycaemic agent

**Table 2:** Baseline and 0–6 month nutrient intake changes in *n* = 242 with complete data during 0–6 months

Nutrient	Median (Q1, Q3)
**Total energy intake**, kJ	7377 (6220, 8619)
**Total energy intake change 0–6 m**, kJ	− 741 (− 1640, − 6)
**Dietary energy density**, kJ/g	6.3 (5.6, 7.1)
**Dietary energy density change 0–6 m**, kJ/g	− 0.3 (− 1.1, 0.4)
**Starches/sugars**, %TEI	43.5 (39.6, 48.2)
**Starches/sugars change 0–6 m**, %TEI	0.6 (− 3.2, 5.4)
**Dietary fibre density**, g/MJ	2.3 (1.9, 2.6)
**Dietary fibre density change 0–6 m**, g/MJ	0.1 (− 0.2, 0.5)
**Total fat**, %TEI	33.8 (30.3, 37.0)
**Total fat change 0–6 m**, %TEI	0.1 (− 3.8, 3.7)
**SFA**, %TEI	11.0 (9.4, 13.0)
**SFA change 0–6 m**, %TEI	0.1 (− 2.2, 1.8)
**MUFA**, %TEI	12.1 (10.5, 13.7)
**MUFA change 0–6 m**, %TEI	0.3 (− 1.5, 2.0)
**PUFA**, %TEI	6.5 (5.3, 7.9)
**PUFA change 0–6 m**, %TEI	0.0 (− 1.6, 1.6)

*Q1* quartile 1, *Q3* quartile 3, *TEI* total energy intake, *SFA* saturated fat, *MUFA* monounsaturated fat, *PUFA* polyunsaturated fat

Weight, BMI, TEI and HbA1c changes were larger in the first compared with the last 6 months of the study. Average weight, BMI, TEI and HbA1c all reduced during 0–6 m (mean change: − 2.3 (95% CI: − 2.7, − 1.8) kg; − 0.8 (− 0.9, − 0.6) kg/m^2^; − 788 (− 953, − 624) kJ; − 1.6 (− 2.6, − 0.6) mmol/mol [− 0.15 (− 0.24, − 0.06) %]). Whilst average weight and BMI remained the same during 6–12 m (0.0 (− 0.3, 0.4) kg and 0.0 (− 0.1, 0.1) kg/m^2^), average TEI and HbA1c reduced further but to a lesser degree (− 365 (− 547, − 184) kJ; − 0.4 (− 1.4, 0.6) mmol/mol [− 0.03 (− 0.13, 0.06) %]).

Missing data analysis (Additional file 1: Table S2) indicated that characteristics of participants in our primary analyses (*n* = 242) differed to the full Early-ACTID cohort (*n* = 593) at baseline by being slightly older (62 vs 61 years), with a lower bodyweight (86.5 vs 89.0 kg) and BMI (29.5 vs 30.4 kg/m^2^), and lowered bodyweight, BMI and HbA1c to a greater degree during 0–6 m (− 2.1 vs − 1.3 kg; − 0.7 vs − 0.5 kg/m^2^; − 2.2 vs − 1.1 mmol/mol [− 0.2 vs − 0.1 %]).

### Pattern 1—‘Carb/fat balance’ dietary pattern

A ‘carb/fat balance’ dietary pattern was identified at baseline. Higher pattern scores correlated with higher percentage energy from starches and sugars (*r* = 0.74) and fibre density (*r* = 0.68) and lower percentage energy from SFA (*r* = − 0.64), MUFA (*r* = − 0.63) and to a lesser extent PUFA (*r* = − 0.12) (Additional file 1: Table S3 [35]). The dietary pattern score thus represented a contrast in the amounts, but not the quality, of carbohydrate and fat intakes. A higher pattern score associated with eating more ‘fruit (fresh)’, ‘low-fat milk’, ‘boiled/baked potatoes’ and ‘legumes’, whilst also eating less ‘higher-fat processed meats’, ‘butter/animal fats’, ‘red meat’ and ‘low-fibre bread’ (Additional file 1: Fig. S1a). A 1-SD higher ‘carb/fat balance’ dietary pattern score at baseline equated to consuming 7.0% more energy from carbohydrate (6.6% from combined starches and sugars and 0.5 g/MJ more fibre) and − 4.9% less energy from fat (− 2.6%, − 2.0%, − 0.3% from SFA, MUFA, and PUFA respectively).

### Pattern 2—‘Obesogenic’ dietary pattern

An energy-dense, higher-fat, lower-fibre (‘obesogenic’) dietary pattern was identified at baseline. Higher scores correlated with higher DED (*r* = 0.81) and percentage energy from fat (*r* = 0.60) and lower fibre-density (*r* = − 0.72) (Additional file 1: Table S4). A higher pattern score associated with eating more ‘low-fibre bread’, ‘processed meat’, ‘coated chicken/fish’ and ‘fried/roast potatoes/chips’, whilst also eating less ‘fruit (fresh)’, ‘vegetables (raw/boiled/grilled)’, ‘yoghurts’ and ‘boiled/baked potatoes’ (Additional file 1: Fig. S1b). A 1-SD higher ‘obesogenic’ dietary pattern score at baseline equated to 1.0 kJ/g higher DED, 3.5% more energy from fat, and 0.4 g/MJ less fibre-density. The ‘obesogenic’ dietary pattern was structurally similar to that previously derived within the UK NDNS (CC = 0.88).

### Changes in dietary patterns

**Fig. 3:**
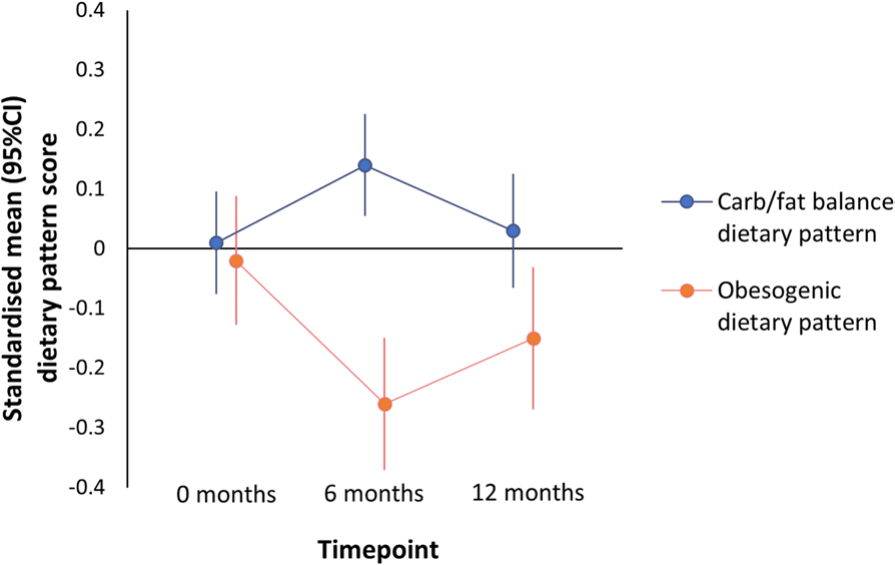
Average standardised diet pattern scores at 0, 6 and 12 months. Pattern scores are offset to aid visualisation

During 0–6 m (*n* = 242), changes in ‘carb/fat balance’ dietary pattern scores indicated movement towards intakes slightly higher in carbohydrates and lower in fats (mean change: 0.12, SD 0.78) (see Fig. 3). In the same period, changes in ‘obesogenic’ dietary pattern scores indicated dietary intakes became less energy-dense, lower-fat and higher-fibre (− 0.24, SD 0.94). During 6–12 m (*n* = 194), dietary patterns reverted back towards intakes lower in carbohydrates and higher in fats (− 0.13, SD 0.81), and to a lesser degree, intakes that were more energy-dense, higher-fat and lower-fibre (0.11, SD 0.96).

Participants who changed their dietary patterns most over 0-6 m had greater reductions in TEI, bodyweight and HbA1c, greater increases in total physical activity and daily minutes of moderate-vigorous physical activity, a higher baseline IMD and were taking more OHA medications (Additional file 1: Table S5-S6). Those who made the greatest change in 'carb/fat balance' dietary pattern score (Additional file 1: Table S5 quintile 5) made slightly greater changes in total carbohydrate, fat and SFA intakes than those who made the greatest change in ‘obesogenic’ dietary pattern score during 0-6 m (Additional file 1: Table S5 quintile 1). Average total carbohydrate, fat and SFA intake in the former at baseline were 41.9%TEI, 36.9%TEI and 12.6%TEI respectively, changing to 49.2%TEI, 32.3%TEI and 10.1%TEI respectively by 6 months.

### Associations between changes in dietary patterns and HbA1c independent of weight changes

**Fig. 4:**
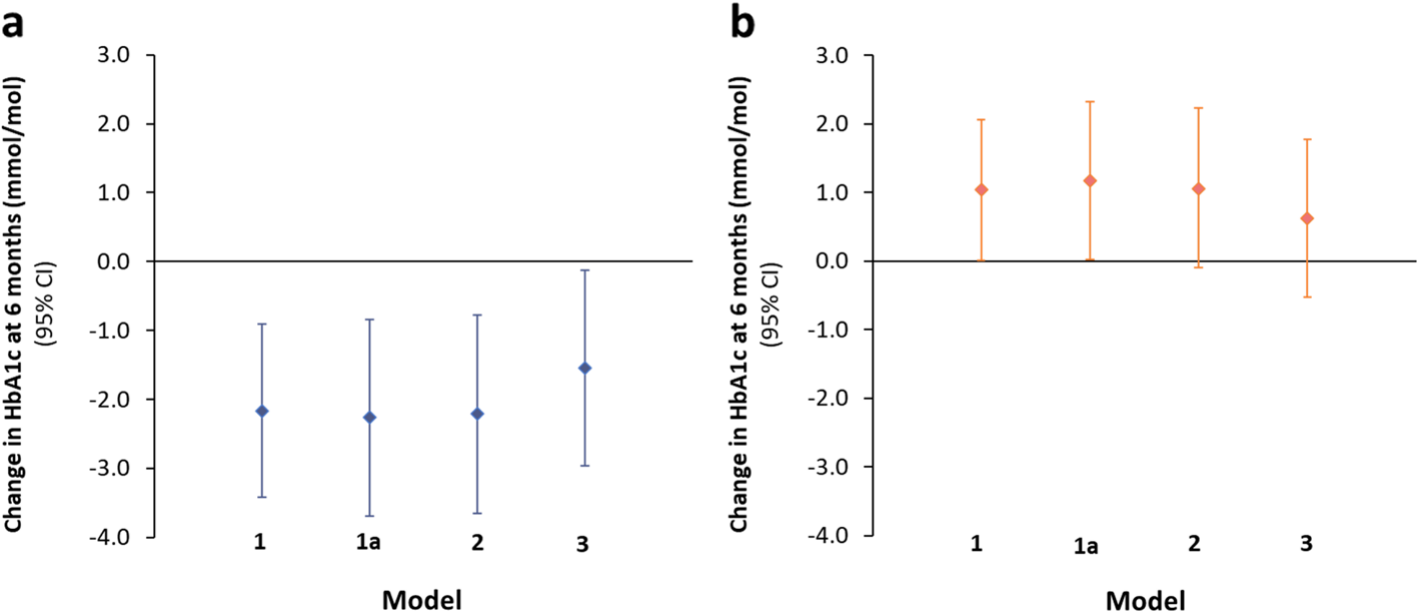
Associations between 1-SD increases in dietary pattern scores during 0–6 months and baseline-adjusted HbA1c at 6 months from multivariable linear regression. **a** Associations between a 1-SD increase in ‘carb/fat balance’ dietary pattern score and HbA1c change at 6 months. **b** Associations between a 1-SD increase in ‘obesogenic’ dietary pattern score and HbA1c change at 6 months. *Model 1a* presents *Model 1* baseline-dietary pattern score adjusted associations in those with complete covariate data. *Model 2* presents associations adjusted for potential confounders: age, sex, baseline under-reporting status and change in total physical activity. *Model 3* presents model 2 associations adjusted for potential mediators: change in bodyweight and energy intake

There was strong evidence that increases in ‘carb/fat balance’ dietary pattern scores were associated with reductions in HbA1c between 0 and 6 m after adjustment for potential confounders (model 2: *β* = − 2.21 [− 3.65, − 0.78] mmol/mol/SD; *p* = 0.003). This association was only partially mediated following further adjustment for bodyweight and TEI change (*β* = − 1.54 [− 2.96, − 0.13] mmol/mol/SD; *p* = 0.033) (Fig. 4a; Additional file 1: Table S7).

We found no evidence of association between changes in ‘obesogenic’ dietary pattern scores and changes in HbA1c after adjustment for potential confounders (0–6 m: *β* = 1.06 [− 0.10, 2.23] mmol/mol/SD; *p* = 0.074) or proposed mediators (*β* = 0.63 [− 0.52, 1.78] mmol/mol/SD; *p* = 0.283) (Fig. 4b; Additional file 1: Table S7).

No evidence associating changes in ‘carb/fat balance’ or ‘obesogenic’ dietary patterns and HbA1c was found after adjustment for potential confounders and mediators during 6–12 m and 0–12 m periods (Additional file 1: Fig. S2; Table S7).

### Sensitivity analyses

Multivariable linear regression analysis provided very strong evidence, regardless of level of adjustment, that reductions in bodyweight associated with the reductions seen in HbA1c (0–6 m: *β* = − 0.70 [95% CI − 0.95, − 0.45] mmol/mol/kg lost; *p* < 0.001) (Additional file 1: Table S8). Additionally, there was strong evidence that both dietary patterns associated with weight change over 0-6 m, with a 1-SD increase in ‘carb/fat balance’ dietary pattern score associating more strongly (*β* = − 1.22 [− 1.89, − 0.55] kg/SD; *p* = < 0.001) than an equivalent 1-SD decrease in ‘obesogenic’ dietary pattern score (*β* = − 0.77 [− 1.31, − 0.23] kg/SD; *p* = 0.006) (Additional file 1: Table S9). Within these models, there was strong evidence that baseline dietary pattern scores also associated with subsequent weight change over 0-6 m, with baseline ‘carb/fat balance’ dietary pattern scores associating more strongly (*β* = − 1.11 [− 1.78, − 0.45] kg per 1-SD higher pattern score; *p* = 0.001) than baseline ‘obesogenic’ dietary pattern scores (*β* = − 0.77 [− 1.31, − 0.23] kg per 1-SD lower pattern score; *p* = 0.005). Higher baseline ‘carb/fat balance’ and lower baseline ‘obesogenic’ dietary pattern scores also associated with greater subsequent reductions in HbA1c within model 2 (*β* = − 1.58 [− 3.01, − 0.14] mmol/mol/SD; *p* = 0.031 and *β* = − 1.18 [− 2.33, − 0.03] mmol/mol/SD; *p* = 0.045 respectively) but not after further adjustment for bodyweight and TEI change (*β* = − 0.92 [− 2.32, 0.49] mmol/mol/SD; *p* = 0.199 and *β* = − 0.69 [− 1.80, 0.43] mmol/mol/SD; *p* = 0.226 respectively).

No evidence was found for interaction between dietary pattern score and sex on HbA1c, or non-linear model trends (Additional file 1: Fig. S3-S4). During 0–6 m, mechanism 1 and 2 effect sizes (*β*) remained largely unattenuated after restricting the sample to only those with complete covariate data (model 1-1a) and upon adjusting for potential confounders (model 1a-2) (Additional file 1: Table S7).

## Discussion

Our study examined whether nutrient-mediated dietary patterns are associated with glycaemic control independent of weight loss in people with type 2 diabetes for the first time. Weight loss was strongly associated with lowering HbA1c. We found evidence that increases in the ‘carb/fat balance’ dietary pattern score associated with reductions in HbA1c, independent of weight loss.

To our knowledge, this is the first study to use RRR to derive distinct dietary patterns theorised to relate to glycaemic control independently or via weight in patients with established type 2 diabetes. Increases in the ‘carb/fat balance’ dietary pattern associated with reductions in HbA1c regardless of initial diet quality or weight loss, in-keeping with our hypothesised nutrient pathway-related mechanisms. Effect sizes per 1-SD ‘carb/fat balance’ dietary pattern change were however small compared to the potential glycaemic benefits associated with weight loss. Weight loss therefore remains key for maximising glycaemic control in type 2 diabetes. The ‘obesogenic’ dietary pattern shared similar nutrient correlations and food group loadings with that seen in previous RRR investigations of obesogenic diets within the UK NDNS [18], indicating that the pattern derived within Early-ACTID was not sample-specific. Although associating with weight change as hypothesised, changes in ‘obesogenic’ dietary pattern scores did not associate with HbA1c change before or after adjusting for weight change. Examining the HbA1c association estimates in our study suggests if the ‘obesogenic’ dietary pattern is associated with HbA1c, it is a smaller association than for the ‘carb/fat balance’ pattern and our sample had insufficient power to confirm it.

Our study participants did not make large dietary pattern changes and were generally consuming what would be considered ‘moderate’ carbohydrate intakes at both 0 and 6 months, averaging 40–46% TEI. Average changes in ‘carb/fat balance’ dietary pattern scores did not, therefore, represent movement towards either ‘high’ or ‘low’ carbohydrate extremes [43]. Caution should therefore be exercised when extrapolating this study’s findings outside of the domains of moderate carbohydrate intakes. Those who increased their ‘carb/fat balance’ dietary pattern score were those who improved HbA1c to the greatest degree and were those found to be moving closer to UK healthy eating guidelines for total carbohydrate (50%TEI), fats (< 35%TEI) and SFA (< 11%TEI) [44–46]. Food group loadings for both dietary patterns we explored also revealed nutrient intake changes coincided with overall higher-quality food choices. Improvements in diet quality have been independently associated with greater cardiometabolic improvements, lower mortality and increased weight loss [47, 48]. Diets prioritising higher-quality food choices yet varying in macronutrient composition, such as Mediterranean-style and vegan diets, have also been associated with improvements in weight and glycaemic control in type 2 diabetes [49, 50]. Evidence from our study suggests that optimal dietary management of type 2 diabetes likely lies in both moderating combined carbohydrate and fat intakes and in maximising overall diet quality.

Our study has many strengths. Glucose-lowering medications were unchanged during 0–6 m, meaning any changes in HbA1c could be attributed solely to lifestyle. Secondly, dietary intakes were assessed using 4-day food diaries, a measure less affected by dietary misreporting compared with other self-report methods [51, 52]. Third, diet measures were repeated at 3 separate timepoints, offering greater detail on effects of the dietary intervention at the primary (6-month) and secondary (12-month) timepoints. Fourth, RRR-derived dietary patterns isolated the best combinations of food intakes that explained intake differences in multiple nutrients hypothesised to relate to glycaemia or bodyweight. This contrasts with alternative methods like principal component analysis (PCA), which captures all variation in diet regardless of disease mechanisms. RRR also has an advantage over a priori scores such as the Mediterranean diet index [53], which may not capture food intakes if such patterns are uncommon in a given population. Finally, analyses were adjusted with high-quality device-measured physical activity measured through accelerometry.

Limitations of this study include potentially biased model estimates due to differential missingness in sample data. Complete data during 0–6 m was available in *n* = 242 (41%) early-ACTID participants. Compared with the whole sample (*n* = 593), our sample were older, had lower baseline bodyweight and HbA1c and saw greater reductions in bodyweight and HbA1c during 0–6 m. Higher values of the HbA1c distribution were thus truncated, potentially downwardly biasing effect estimates. Secondly, the dietary pattern that explained maximal variation in carbohydrate and fat intakes associated with total amounts of these nutrients, regardless of quality (i.e. both sugar and fibre were higher), and was unable to capture differences in PUFA to as great a degree as other fats. We were thus unable to answer questions about whether patterns differentiating carbohydrate and fat quality associated with HbA1c change, as these were not the changes that participants made. Third, participants with lower quality diets at baseline were found to be those who improved dietary pattern scores the most, and vice-versa, suggesting that extremes of dietary pattern score changes represent potential regression to the mean. However, we constructed models to include dietary pattern score at baseline so that change in our adjusted models represented a 1-SD increase in pattern score when people start from the same point. Fourth, we identified 56% of our sample as energy under-reporters, in line with under-reporting observed in adults in the UK NDNS [36]. This highlights a potentially high degree of error; however, under-reporting was not associated with dietary pattern score and regression estimates were not attenuated by misreporting adjustment. Fifth, due to demographic characteristics in Early-ACTID participants, study results are mainly generalisable to a white population living in less socially deprived areas. Finally, although we adjusted for a number of potentially confounding variables, as with all observational analyses, residual confounding from unmeasured confounders cannot be ruled out.

## Conclusions

In newly diagnosed people with type 2 diabetes, promoting weight loss should remain the primary nutritional strategy for improving glycaemic control. However, improvements in dietary pattern quality that bring carbohydrate and fat intakes more in line with general healthy eating targets may provide small, additional improvements in HbA1c.

## Supplementary Information

**Additional file 1: Supplementary information S1.** Further details on dietary misreporting calculation. **Supplementary information S2.** Further details on sensitivity analyses. **Supplementary information S3.** Code used for employing reduced-rank regression in Stata (via SAS). **Table S1.** Food groups and their contents for the ‘obesogenic’ and ‘carb/fat balance’ dietary patterns. **Table S2.** Characteristics of all Early-ACTID participants compared with participants with complete covariate data for adjusted secondary analyses (periods 6-12m and 0-12m). **Table S3.** Explained nutrient variation and correlations for ‘carb/fat balance’ dietary patterns derived at 0, 6 and 12 months. **Table S4.** Explained nutrient variation and nutrient correlations for energy-dense, higher-fat, lower-fibre ‘obesogenic’ dietary patterns derived at 0, 6 and 12 months. **Table S5.** Descriptive dietary and sample characteristics in extreme quintiles of ‘carb/fat balance’ dietary pattern score change during 0-6m. **Table S6.** Descriptive dietary and sample characteristics in extreme quintiles of ‘obesogenic’ dietary pattern score change during 0-6m. **Table S7.** Relation between change in HbA1c and change in dietary pattern scores during study periods. **Table S8.** Relation between change in HbA1c and change in bodyweight during study periods. **Table S9.** Relation between change in bodyweight and change in dietary pattern score during study periods. **Table S10.** Relation between change in HbA1c and change in dietary pattern scores during study periods, with additional adjustment for trial arm in models 2-3. **Fig. S1.** Dietary pattern factor loading diagrams. **Fig. S2.** Associations between 1-SD increases in dietary pattern scores during 6-12 or 0-12 month periods and start-of-period-adjusted HbA1c at 12 months from multivariable linear regression. **Fig. S3.** Fitted lines by sex from simple linear regression analyses of end-of-period HbA1c on ‘carb/fat balance’ and ‘obesogenic’ dietary pattern change, to investigate potential interactions between dietary pattern change and sex. **Fig. S4.** Linear trend assessment.

## Data Availability

Data supporting the findings of this study are not openly available due to consent not being sought at the time of the trial. However, authenticated researchers may apply for access to the data via the University of Bristol data.bris research repository (DOI: 10.5523/bris.3o7bip8v2ae8m2gdfpu1pt5rlz).

## Abbreviations

BMI: Body mass index
CC: Congruence coefficient
CI: Confidence interval
DED: Dietary energy density
Early-ACTID: Early ACTivity-In-Diabetes (trial)
IMD: Index of Multiple Deprivation
MUFA: Monounsaturated fatty acids
MVPA: Moderate-vigorous physical activity
NDNS: National diet and Nutrition Survey
OHA: Oral hypoglycaemic agents
PUFA: Polyunsaturated fatty acids
Q1: Quartile 1
Q3: Quartile 3
RRR: Reduced-rank regression
SD: Standard deviation
SFA: Saturated fatty acids
T2D: Type 2 diabetes
TEI: Total energy intake
