# Supplementary material for: Is glycaemic control associated with dietary patterns independent of weight change in people newly diagnosed with type 2 diabetes? Prospective analysis of the Early-ACTivity-In-Diabetes trial
Source: BMC Med. 2022 Apr 18;20:161. doi: 10.1186/s12916-022-02358-5 (PMC9014614; doi:10.1186/s12916-022-02358-5)

# Additional file 1 - Supplementary Materials

**Supplementary information S1**: Further details on dietary misreporting calculation

PAL categories were assigned based on baseline minutes of moderate-vigorous physical activity (MVPA) [38]. A 1-SD cut-off [39] around reported energy intake/estimated energy requirement (rEI/EER) (20% in men and 19% in women) was used assuming energy balance (rEI/EER=1.0) to allocate misreporting status as plausible (e.g. rEI/EER=0.8 to 1.2), under- (e.g. rEI/EER<0.80) or over-reporting (e.g. rEI/EER>1.20).

**Supplementary information S2**: Further details on sensitivity analyses

**In *M****odel 1a*, we estimated the impact of having missing covariate data on the unadjusted estimate in model 1 by repeating the model in a restricted sample that had complete data on all covariates included in models 2-3. The **effects of missing data versus confounder adjustment were differentiated by comparing changes in the β and 95% CI in models 1 to 1a (missing data effect i.e. same adjustment in a smaller sample) and models 1a to 2 (confounder adjustment effect i.e. different adjustment in the same sample). To estimate missing data bias in our analyses, we examined differences in sample characteristics owing to missing data on diet and covariates by comparing participants with complete data against the full Early-ACTID sample during each period.**

To test assumptions of linearity, **tests for trend between dietary pattern score change quintiles and their estimated marginal (HbA1c) means during each period were performed using a likelihood ratio test. This compared goodness of fit of modelling categorical (quintiles of) dietary pattern score change versus continuous score change in model 2. Potential interactions between dietary pattern and sex were also explored by including an interaction term between dietary pattern score change and sex in model 2.**

We believed including trial arm in our main analysis models would lead to model over-adjustment. The trial arm that participants had been assigned to was considered to lie earlier on the causal pathway potentially linking dietary change to HbA1c change, meaning it would not be confounding relationships between diet and HbA1c. Additionally, other targets of the intervention such as physical activity, energy intake and bodyweight changes were to be separately adjusted for within our models. However, to explore any effects of potential confounding by trial arm, models 2-3 were repeated with trial arm adjustment included.

To confirm relationships between weight change and HbA1c, we repeated models 1-2 for each period. *Model 1* estimated unadjusted associations between period weight change (exposure) and end-of-period HbA1c (outcome), adjusting for start-of-period HbA1c and weight. *Model 2* estimated associations independent of potential confounders by adjusting for age, sex, and period-change in total physical activity. Finally, we estimated associations of changes in dietary patterns (exposure) with end-of-period bodyweight (outcome) between 0-6m, 6-12m, or 0-12m (outcomes) using models 1-2 outlined above but adjusting for start-of-period weight instead of HbA1c. *Model 3* in these cases estimated mediation by adding TEI only.

**Supplementary information S3**: Code used for employing reduced-rank regression in Stata (via SAS).

**Reduced Rank Regression of percentage energy from combined starches and sugars (percent_cho), fibre density (fd), percentage energy from saturated fat (percent_sfa), percentage energy from monounsaturated fat (percent_mufa), and percentage energy from polyunsaturated fat (percent_pufa) with all food groups (ave_grams_foodgroup1-ave_grams_foodgroup65)**

plssas, y(percent_cho fd percent_sfa percent_mufa percent_pufa) x(ave_grams_foodgroup1-ave_grams_foodgroup65) method(rrr)

exe(C:\Program Files\SASHome\SASFoundation\9.4\sas.exe)

insheet using "N:\Stata output\out.csv", comma clear

save “PersonLevelFile_earlyactid_direct_RRR.dta", replace

**Reduced Rank Regression of dietary energy density (ded), fibre density (fd) and percentage energy from fat (percent_fat) with all food groups (ave_grams_foodgroup1-ave_grams_foodgroup47)**

plssas, y(ded fd percent_fat) x(ave_grams_foodgroup1-ave_grams_foodgroup47) method(rrr)

exe(C:\Program Files\SASHome\SASFoundation\9.4\sas.exe)

insheet using "N:\Stata output\out.csv", comma clear

save “PersonLevelFile_earlyactid_indirect_RRR.dta", replace

**Table S1*:*** Food groups and their contents for the ‘obesogenic’ and ‘carb/fat balance’ dietary patterns.

| **Obesogenic dietary pattern** | |  | **Carb/fat balance dietary pattern** | | | |
| --- | --- | --- | --- | --- | --- | --- |
| **Food group** | **Contents** |  | **Food group** | | **Contents** | |
| **Alcoholic drinks** | Beer, cider, wine and spirits |  | | **Alcoholic drinks** | | Beer, cider, wine and spirits |
| **Biscuits and cakes** | Sweet biscuits (all types) including chocolate-covered, sweet buns, cakes and pastries (all sweet types), cereal bars |  | | **Biscuits (sweet)** | | Sweet biscuits (all types) including chocolate-covered |
|  |  |  | | **Cakes** | | Sweet buns, cakes and pastries (all sweet types), cereal bars |
| **Butter and animal fats** | Full-fat and half-fat butter |  | | **Butter and animal fats** | | Full-fat and half-fat butter, palm oil, vegetable suet |
| **Cereal based mixed meals** | Pasta in sauce, pasta with cheese, pasta and rice salads, tabbouleh, risotto |  | | **Cereal based mixed meals** | | Pasta in sauce, pasta with cheese, pasta and rice salads, tabbouleh, risotto, egg-fried rice |
| **Cheese** | Cheeses and cheese spreads, including cottage cheese |  | | **Cottage cheese** | | Reduced-fat and full-fat cottage cheese: plain or with additions |
|  |  |  | | **Other cheese** | | All cheese and cheese spreads other than cottage cheese |
| **Chocolate and confectionery** | Plain chocolate and chocolate-based confectionary, table sugar, syrups, sweets |  | | **Chocolate** | | Plain chocolate and chocolate-based confectionary |
| **Spreads (sweet)** | Honey, fruit-jams and curds |  | | **Spreads (sweet) and confectionery** | | Honey, fruit-jams and curds, table sugar, syrups, sweets |
| **Coated chicken and fish** | Fish or chicken in batter or breadcrumbs |  | | **Coated chicken and fish** | | Fish or chicken in batter or breadcrumbs |
| **Condiments** | Herbs, spices, stock cubes |  | | **Condiments** | | Herbs, spices, stock cubes |
| **Sauces (low energy density < 10 kJ/g)** | Water-, milk-, cheese-, vinegar-, vegetable-, or fruit-based sauces and dressings |  | | **Sauces (low energy density < 10 kJ/g)** | | Water-, milk-, cheese-, vinegar-, vegetable-, or fruit-based sauces and dressings |
| **Boiled/baked potatoes** | Boiled, mashed and baked potatoes, gnocchi |  | | **Boiled/baked potatoes** | | Boiled and baked potatoes, gnocchi |
| **Crisps and savoury snacks** | Potato, wheat-based, vegetable and corn crisps, pretzels, papadums |  | | **Crisps and savoury snacks** | | Potato, wheat-based, vegetable and corn crisps, pretzels, papadums |
| **Eggs and egg dishes** | Boiled/poached/fried/scrambled eggs, omelettes, quiches, egg-fried rice, egg mayonnaise, Scotch eggs |  | | **Eggs** | | Boiled/ poached/dry-fried/plain-scrambled eggs |
|  |  |  | | **Egg dishes** | | Egg mayonnaise, omelettes, fried and scrambled eggs |
| **Fish** | Fish; canned, boiled, steamed, poached, baked or grilled; plain or in pies or pastry, curry; not battered or breaded |  | | **White fish and shellfish** | | Non-oily fish; canned, boiled, steamed, poached, baked or grilled; not battered or breaded |
|  |  |  | | **Oily fish** | | Canned, grilled, baked, or fried anchovies, herring, kipper, mackerel, pilchards, salmon, sardines, trout; not battered or breaded |
|  |  |  | | **Fish and shellfish mixed dishes** | | Fisherman's pie, tuna mayonnaise, fish or shellfish curries, in sauce, pasta. |
| **Fruit (fresh)** | Fresh fruit, including canned in juice |  | | **Fruit (fresh)** | | Fresh fruit, including canned in juice |
| **Fruit (other)** | Stewed (with or without sugar), baked, dried, and canned in syrup |  | | **Fruit (other)** | | Dried fruit (all types) |
| **Fried/roast vegetables** | Fried or roasted vegetables, excluding potatoes |  | | **Fried/roast vegetables** | | Fried or roasted vegetables, excluding potatoes |
| **Fried/roast potatoes and chips** | Fried or roast potatoes, chips, potato waffles |  | | **Fried/roast, chips and other potato with added fat** | | Fried, mashed or roast potatoes, chips, potato waffles, potato salad (with mayonnaise), cheese and potato 'pie' |
| **Fruit juice** | Fruit juice (all types), fruit smoothies |  | | **Fruit juice** | | Fruit juice (all types), fruit smoothies |
| **High fat milk and cream** | Fresh or powdered whole cow's or goat's milk, milkshakes, cream (all types), crème fraiche, evaporated/condensed milk |  | | **High fat milk** | | Fresh or powdered whole cow's or goat's milk, milkshakes |
|  |  |  | | **Cream** | | Cream (all types), crème fraiche, evaporated/condensed milk |
| **High fibre bread** | Brown, wholemeal, granary, rye, white (with added fibre) bread or rolls |  | | **High fibre bread** | | Brown, wholemeal, granary, rye, white (with added fibre) bread or rolls |
| **High fibre breakfast cereals** | Bran- or wheat-based cereals, oatmeal/porridge, muesli |  | | **High fibre breakfast cereals** | | Bran- or wheat-based cereals, oatmeal/porridge; excluding muesli |
|  |  |  | | **Muesli** | | Muesli (all types) |
| **Hot and powdered drinks** | Teas, coffees, malted drinks, hot chocolate, diet powder drinks |  | | **Powdered drinks** | | Instant coffees, malted drinks, hot chocolate |
| **Low energy drinks** | Fruit squashes or cordials and carbonated drinks with sweetener/no sugar added |  | | **Low energy drinks (hot and cold)** | | Fruit squashes or cordials and carbonated drinks with sweetener/no sugar added, black tea, black coffee, water |
| **Water** | Mineral, flavoured and tap water |  | |  | |  |
| **Ice-creams** | All ice-cream-based desserts |  | | **Ice-creams** | | All ice-cream-based desserts |
| **Legumes** | Boiled, canned, baked-in-sauce beans and lentils |  | | **Legumes** | | Boiled, canned, baked-in-sauce beans, peas and lentils |
| **Low fat milk** | Skimmed/semi-skimmed fresh or powdered dairy milk, soya milk, powdered hot drinks made up with skimmed/semi-skimmed milk |  | | **Low fat milk** | | Skimmed/semi-skimmed fresh or powdered dairy milk, soya milk, powdered hot drinks made up with skimmed/semi-skimmed milk |
| **Low fibre bread** | White bread, bagels, breadcrumbs, rolls and tortillas |  | | **Low fibre bread** | | White bread, bagels, breadcrumbs, rolls and tortillas |
| **Margarine and Vegetable oils** | PUFA/non-PUFA spreads, cod liver oil, seed oils, palm oil, vegetable suet |  | | **PUFA oils** | | Vegetable oil, sunflower oil, cod liver oil |
|  |  |  | | **MUFA oils** | | Olive oil, rapeseed oil |
|  |  |  | | **PUFA spreads** | | Polyunsaturated spreads and margarines (35-70% fat), flora proactive |
|  |  |  | | **Other spreads** | | Non-PUFA-based spreads and margarines |
| **Meat and poultry** | Beef, lamb, pork, chicken, duck, offal; stewed, fried, grilled or roasted |  | | **Red meat** | | Beef, lamb, pork, duck, game with fat; stewed, fried, grilled or roasted |
|  |  |  | | **Lean red meat** | | Offal, lean beef, lamb or pork; fried, grilled or roasted |
|  |  |  | | **Poultry** | | Chicken or turkey; stewed, fried or roasted |
| **Meat mixed dishes** | Meat and poultry in casseroles, curries, stews, Bolognese, lasagne, pies and pastries |  | | **Poultry mixed dishes** | | Chicken/turkey casserole, curry/in sauce, Bolognese; excluding soups |
|  |  |  | | **Red meat mixed dishes** | | Beef, lamb, pork, or offal curries, stews, hotpots, Bolognese, lasagnes or cooked with vegetables; excluding soups. |
|  |  |  | | **Savoury pies/pastries** | | Vegetable- or meat-based pies and pastries, including plain pastry and savoury suet pudding |
| **Meat substitutes** | Quorn, Soya mince, tofu |  | | **Meat substitutes** | | Quorn, Soya mince, tofu |
| **Nuts and seeds** | Nuts and seeds (all types) including coconut flesh |  | | **Nuts and seeds** | | Nuts and seeds (all types) including coconut flesh |
| **Other bread products** | Breads with added fat such as brioche, naan, garlic and fried. Dumplings, stuffing, Yorkshire puddings, savoury biscuits, bran, plain pastry |  | | **Other bread products** | | Breads with added fat such as brioche, naan, garlic and fried. Dumplings, stuffing, Yorkshire puddings |
|  |  |  | | **Biscuits (savoury)** | | Breadsticks, crackers, crispbreads, oatcakes and rice cakes |
| **Low fibre breakfast cereals** | Corn flakes, Frosties, Rice Krispies, Special K |  | | **Low fibre breakfast cereals** | | Corn flakes, Frosties, Rice Krispies, Special K |
| **Pizza** | Pizza (all types) |  | | **Pizza** | | Pizza (all types) |
| **Processed meat** | Bacon, gammon, ham, burgers, corned beef, sausages, meat spreads and paté, cured meats |  | | **Lower fat processed meats** | | Lean bacon, gammon, ham, reduced-fat pork sausages, low-fat meat spreads and patés, turkey ham |
|  |  |  | | **Higher fat processed meats** | | Bacon, burgers, corned beef, sausages, meat spreads, full-fat pâté, cured meats; with no fat removed |
|  |  |  | | **Processed meat mixed meals** | | Baked beans with sausages, burgers in buns, corned beef hash, scotch eggs, sausage casserole, toad in the hole |
| **Puddings** | Sweet pies, crumbles, tarts, meringues, cheesecakes, custards, pancakes, mousses, rice puddings, jellies, sponge puddings |  | | **Puddings** | | Sweet pies, crumbles, tarts, meringues, cheesecakes, custards, pancakes, mousses, rice puddings, jellies, sponge puddings, stewed fruit, fruit canned in syrup |
| **Rice, pasta and other grains** | White or brown rice, pasta, couscous, noodles, pearl barley, quinoa |  | | **White rice, pasta and other grains** | | White rice, pasta, couscous, noodles, pearl barley |
|  |  |  | | **Whole-grain rice, pasta and other grains** | | Wheat-bran, brown rice, pasta, quinoa |
| **Sauces (higher energy density > 10 kJ/g)** | Egg-, cream-, or oil-based sauces |  | | **Sauces (higher energy density > 10 kJ/g)** | | Egg-, cream-, or oil-based sauces |
| **Soups** | Soup (all types except stews) |  | | **Soups** | | Soup (all types except stews) |
| **Reduced/sugar-free confectionery** | Sugar-free/reduced-sugar sweets and chocolates, artificial sweeteners |  | | **Reduced/sugar-free confectionery** | | Sugar-free/reduced-sugar sweets, chocolates and sweet spreads, artificial sweeteners |
| **Sugar-sweetened drinks** | Fruit squashes or cordials and carbonated drinks with added sugar, energy drinks |  | | **Sugar-sweetened drinks** | | Fruit squashes or cordials and carbonated drinks with added sugar, energy drinks, diet powder drinks |
| **Vegetable mixed dishes** | Mixed vegetables in cheese-based sauces, curries, casseroles, chilli’s, lasagne, pie's, flans, pastry, pâtés, falafel, guacamole, mayonnaise salads; excluding soups |  | | **Vegetable mixed dishes** | | Mixed vegetables in cheese-based sauces, curries, casseroles, chilli’s, lasagne, shepherd's pie, pâtés, falafel, guacamole; mayonnaise salads; excluding soups |
| **Vegetables (Raw/boiled/grilled)** | Raw, pickled, grilled or boiled vegetables, avocado, peas |  | | **Vegetables (Raw/boiled/grilled)** | | Raw, pickled, grilled or boiled vegetables, avocado |
| **Yoghurts** | Plain and flavoured dairy/soya yoghurts, fromage frais, probiotic/drinking yoghurts |  | | **Plain yoghurts** | | Plain dairy/soya yoghurt, fromage frais |
|  |  |  | | **Other yoghurts** | | Flavoured/fruit yoghurts and fromage frais, probiotic/drinking yoghurts |

**Table S2**: Characteristics of all Early-ACTID participants compared with participants with complete covariate data for adjusted secondary analyses (periods 6-12m and 0-12m*)*. Data marked in bold relates to variables used in analysis models for that period. Data presented as n (%) or median (Q1, Q3).

|  |  | **n** | **Early-ACTID participant characteristics** | **n** | **6-12m participant characteristics with complete covariate data (models 1a-3)** | **n** | **0-12m participant characteristics with complete covariate data (models 1a-3)** |
| --- | --- | --- | --- | --- | --- | --- | --- |
| **n** (%) |  |  | 593 (100%) |  | 194 (100%) |  | 214 (100%) |
| **Arm**, n (%) |  |  |  |  |  |  |  |
|  | Usual care | 593 | 99 (17%) | **194** | **9 (5%)** | **214** | **22 (10%)** |
|  | Diet | 593 | 248 (42%) | **194** | **93 (48%)** | **214** | **95 (44%)** |
|  | Diet & Exercise | 593 | 246 (41%) | **194** | **92 (47%)** | **214** | **97 (45%)** |
| **Male**, n (%) |  | 593 | 383 (65%) | **194** | **136 (70%)** | **214** | **151 (71%)** |
| **White ethnicity**, n (%) |  | 593 | 567 (96%) | 194 | 189 (97%) | 214 | 210 (98%) |
| **Smoker at 0 months**, n (%) |  | 593 | 48 (8%) | 194 | 13 (7%) | 214 | 14 (7%) |
| **Age at 0 months**, years |  | 592 | 61 (53, 68) | **194** | **63 (58, 69)** | **214** | **63 (57, 69)** |
| **Time since diagnosis at 0 months**, years |  | 592 | 0.5 (0.4, 0.6) | 194 | 0.5 (0.4, 0.6) | 214 | 0.5 (0.4, 0.6) |
| **IMD score at 0 months** |  | 591 | 12.7 (7.2, 20.0) | 193 | 12.6 (6.3, 19.1) | 213 | 12.6 (6.5, 19.1) |
| **Total activity at 0 months**, counts/min |  | 546 | 286 (218, 366) | 184 | 293 (222, 369) | **214** | **291 (221, 367)** |
| **Total activity at 6 months**, counts/min |  | 495 | 298 (218, 395) | **194** | **332 (226, 409)** | 200 | 330 (223, 406) |
| **Total activity change 0-6m**, counts/min |  | 461 | 9 (-44, 75) | 184 | 22 (-42, 93) | 200 | 10 (-43, 86) |
| **Total activity change 6-12m**, counts/min | | 415 | -4 (-61, 49) | **194** | **-3 (-72, 45)** | 200 | -1 (-71, 47) |
| **Total activity change 0-12m**, counts/min | | 429 | 2 (-51, 67) | 184 | 2 (-49, 81) | **214** | **2 (-52, 78)** |
| **MVPA at 0 months**, mins/day |  | 546 | 21 (12, 36) | 184 | 23 (13, 37) | **214** | **22 (11, 37)** |
| **MVPA at 6 months**, mins/day |  | 495 | 25 (12, 43) | **194** | **32 (15, 48)** | 200 | 30 (14, 48) |
| **MVPA change 0-6m**, mins/day |  | 461 | 2 (-7, 11) | 184 | 3 (-5, 19) | 200 | 2 (-7, 18) |
| **MVPA change 6-12m**, mins/day |  | 415 | -1 (-9, 6) | **194** | **-1 (-11, 5)** | 200 | -1 (-10, 5) |
| **MVPA change 0-12m**, mins/day |  | 429 | 0 (-8, 12) | 184 | 1 (-9, 15) | **214** | **0 (-8, 14)** |
| **Weight at 0 months**, kg |  | 592 | 89.0, (80.0, 99.3) | 194 | 86.1 (77.9, 94.1) | **214** | **86.9 (78.6, 95.7)** |
| **Weight at 6 months**, kg |  | 579 | 87.1 (77.7, 98.4) | **194** | **83.9 (75.2, 92.0)** | 214 | 84.6 (75.8, 94.1) |
| **Weight change 0-6m**, kg |  | 578 | -1.3 (-3.4, 0.6) | 194 | -2.1 (-4.0, -0.2) | 214 | -2.1 (-3.8, -0.2) |
| **Weight change 6-12m**, kg |  | 562 | 0.3 (-1.2, 2.0) | **194** | **0.1 (-1.3, 1.8)** | 214 | 0.2 (-1.4, 1.9) |
| **Weight change 0-12m**, kg |  | 564 | -0.9 (-3.6, 1.3) | 194 | -1.8 (-5.1, 0.2) | **214** | **-1.5 (-4.8, 0.8)** |
| **BMI at 0 months**, kg/m2 |  | 592 | 30.4 (27.8, 34.2) | 194 | 29.3 (27.3, 32.6) | **214** | **29.3 (27.3, 33.0)** |
| **BMI at 6 months**, kg/m2 |  | 579 | 29.6 (27.2, 33.6) | **194** | **28.6 (26.4, 32.0)** | 214 | 28.7 (26.5, 32.4) |
| **BMI change 0-6m**, kg/m2 |  | 578 | -0.5 (-1.2, 0.2) | 194 | -0.7 (-1.4, -0.1) | 214 | -0.7 (-1.3, -0.1) |
| **BMI change 6-12m**, kg/m2 |  | 562 | 0.1 (-0.4, 0.7) | **194** | **0.0 (-0.5, 0.6)** | 214 | 0.1 (-0.5, 0.7) |
| **BMI change 0-12m**, kg/m2 |  | 564 | -0.3 (-1.2, 0.4) | 194 | -0.6 (-1.8, 0.1) | **214** | **-0.5 (-1.6, 0.3)** |
| **HbA1c at 0 months**, mmol/mol |  | 593 | 48 (43, 54) | 194 | 48 (43, 54) | **214** | **48 (43, 54)** |
| **HbA1c at 0 months**, % |  | 593 | 6.5 (6.1, 7.1) | 194 | 6.5 (6.1, 7.1) | **214** | **6.5 (6.1, 7.1)** |
| **HbA1c at 6 months**, mmol/mol |  | 569 | 47 (42, 53) | **194** | **45 (41, 51)** | 213 | 45 (41, 51) |
| **HbA1c at 6 months**, % |  | 569 | 6.4 (6.0, 7.0) | **194** | **6.3 (5.9, 6.8)** | 213 | 6.3 (5.9, 6.8) |
| **HbA1c change 0-6m**, mmol/mol |  | 569 | -1.1 (-4.4, 3.3) | 194 | -2.2 (-6.6, 1.1) | 213 | -2.2 (-5.5, 2.2) |
| **HbA1c change 0-6m**, % |  | 569 | -0.1 (-0.4, 0.3) | 194 | -0.2 (-0.6, 0.1) | 213 | -0.2 (-0.5, 0.2) |
| **HbA1c change 6-12m**, mmol/mol |  | 560 | 1.1 (-2.2, 3.3) | **194** | **0.0 (-2.2, .2)** | 213 | 0.0 (-2.2, 2.2) |
| **HbA1c change 6-12m**, % |  | 560 | 0.1 (-0.2, 0.3) | **194** | **0.0 (-0.2, 0.2)** | 213 | 0.0 (-0.2, 0.2) |
| **HbA1c change 0-12m**, mmol/mol |  | 574 | -1.1 (-4.4, 4.4) | 194 | -2.2 (-6.0, 1.1) | **214** | **-2.2 (-6.0, 2.2)** |
| **HbA1c change 0-12m**, % |  | 574 | -0.1 (-0.4, 0.4) | 194 | -0.2 (-0.5, 0.1) | **214** | **-0.2 (-0.5, 0.2)** |
| **OHA prescription at 0 months**, n (%) |  |  |  |  |  |  |  |
|  | Metformin | 593 | 206 (35%) | 194 | 66 (34%) | 214 | 74 (35%) |
|  | Sulphonylurea | 593 | 50 (8%) | 194 | 15 (8%) | 214 | 18 (8%) |
|  | Glitazone | 593 | 7 (1%) | 194 | 2 (1%) | 214 | 2 (1%) |
| **OHA prescription from 6 months**, n (%) |  |  |  |  |  |  |  |
|  | Metformin | 593 | 209 (35%) | 194 | 66 (34%) | 214 | 74 (35%) |
|  | Sulphonylurea | 593 | 53 (9%) | 194 | 15 (8%) | 214 | 19 (9%) |
|  | Glitazone | 593 | 9 (2%) | 194 | 2 (1%) | 214 | 3 (1%) |

*IMD – index of multiple deprivation; MVPA – moderate-vigorous physical activity; OHA - oral-hypoglycaemic agent.*

**Table S3**: Explained nutrient variation and correlations for ‘carb/fat balance’ dietary patterns derived at 0, 6 and 12-months.

|  |  |  |  | **Explained variation (%)** | | | |  |  | |  | |  |  |  | **Correlation coefficient** | | |  |  | |
| --- | --- | --- | --- | --- | --- | --- | --- | --- | --- | --- | --- | --- | --- | --- | --- | --- | --- | --- | --- | --- | --- |
| **Timepoint** | **Nutrient intake (total)** | **Starches and sugars (%)** | | | **Fibre density (g/MJ)** | **SFA (%)** | **MUFA (%)** | | | **PUFA (%)** | | **Food intake (total)** |  | **Starches and sugars (%)** | | | **Fibre density (g/MJ)** | **SFA (%)** | **MUFA (%)** | | **PUFA (%)** |
| **0 months** | 36.2 | 53.9 | | | 45.1 | 40.9 | 39.9 | | | 1.4 | | 2.1 |  | 0.74 | | | 0.68 | -0.64 | -0.63 | | -0.12 |
| **6 months** | 39.2 | 57.5 | | | 47.4 | 45.7 | 44.2 | | | 1.1 | | 2.4 |  | 0.76 | | | 0.69 | -0.68 | -0.67 | | -0.10 |
| **12 months** ^a^ | 40.0 | 57.0 | | | 45.2 | 44.5 | 51.1 | | | 2.3 | | 2.7 |  | -0.75 | | | -0.67 | 0.67 | 0.71 | | 0.15 |

*SFA - saturated fat; MUFA - mono-unsaturated fat; PUFA - poly-unsaturated fat.*

^a^ A congruence coefficient between dietary patterns at separate timepoints of ≥0.95, 0.85-0.94, or <0.85 suggest good, fair, or unacceptable similarity respectively [35]. We calculated a congruence coefficient of 0.89 between 0- and 6-month dietary patterns. However, a negative congruence coefficient was obtained for 0 and 12-month dietary patterns (congruence coefficient: -0.87). This coincided with equal in magnitude, but opposing in sign, dietary pattern-nutrient correlations at these timepoints. Given ‘fair’ pattern structural similarity, the independently derived 12-month dietary pattern scores were interpreted as essentially being ‘inverted’ baseline pattern scores as an artefact of the reduced-rank regression’s final iteration. Multiplying all food group pattern loadings at 12 months by -1 moved the direction of association of the dietary pattern score with its nutrient response variables to be in line with those seen at 0 months. Projecting this single, 0-month dietary pattern structure onto 6 and 12-month data thus allowed for a meaningful analysis of changes in a single dietary pattern score over time.

**Table S4**: Explained nutrient variation and nutrient correlations for energy-dense, higher-fat, lower-fibre ‘obesogenic’ dietary patterns derived at 0, 6 and 12 months. Congruence coefficients equalled 0.90 between 0-months and both 6- and 12-month dietary patterns.

|  |  |  | | **Explained variation (%)** | | | |  |  | **Correlation coefficient** | | |  |
| --- | --- | --- | --- | --- | --- | --- | --- | --- | --- | --- | --- | --- | --- |
| **Timepoint** | **Nutrient intakes (total)** | | **DED (g/kJ)** | | **Fat (%)** | **Fibre density (g/MJ)** | **Food intake (total)** |  | **DED (g/kJ)** | | **Fat (%)** | **Fibre density (g/MJ)** | |
| **0 months** | 51.3 | | 66.7 | | 35.9 | 51.2 | 3.4 |  | 0.81 | | 0.60 | -0.72 | |
| **6 months** | 58.1 | | 73.3 | | 44.4 | 56.5 | 3.6 |  | 0.86 | | 0.67 | -0.75 | |
| **12 months** | 58.1 | | 70.4 | | 47.0 | 56.9 | 3.8 |  | 0.84 | | 0.69 | -0.75 | |

*DED – dietary energy density.*

**Table S5**: Descriptive dietary and sample characteristics in extreme quintiles of ‘carb/fat balance’ dietary pattern score change during 0-6m. Participants in quintile 5 made the greatest change towards higher-carb, lower-fat intakes during 0-6 months (mean change: 1.25, SD 0.42). Participants in quintile 1 made the least dietary change in this direction (mean change: -0.91, SD 0.46; suggesting average participant dietary intakes in this quintile became lower in carbohydrate and higher in fat compared to baseline intakes). All other data presented as n (%) or median (Q1, Q3). Highest loading food group intakes in median (Q1, Q3) g/day are presented to indicate the average amounts (and change in amounts) of foods consumed. Percentage of consumers (and change in consumers) of these food groups indicate the number of (and change in number of) individuals consuming any amount of these foods. Median ‘Meat substitutes’ intake is zero, for example, but should be interpreted in context of percentage of consumers being very low (2% of whole sample).

| **Highest and lowest quintile sample characteristics (models 1a-3) of 0-6m ‘carb/fat balance’ dietary pattern score change** | | | | |
| --- | --- | --- | --- | --- |
|  | **Whole sample** | **Quintile 1** | **Quintile 5** |  |
| **n** | 242 | 49 | 48 |  |
| **Carb/fat balance dietary pattern score at baseline**, SD | 0.04±0.79 | 0.66±0.72 | -0.67±0.69 |  |
| **Carb/fat balance dietary pattern score change 0-6m**, SD | 0.12±0.78 | -0.91±0.46 | 1.25±0.42 |  |
| **TEI at baseline**, kJ | 7347 (6220, 8619) | 7464 (6166, 8413) | 8055 (6770, 9170) |  |
| **TEI change 0-6m**, kJ | -731 (-1647, -6) | -367 (-1640, 54) | -1078 (-1922, -445) |  |
| **Starches and sugars at baseline**, %TEI | 43.5 (39.8, 48.1) | 46 (42.7, 51.2) | 40.3 (35.6, 43.2) |  |
| **Starches and sugars change 0-6m**, %TEI | 0.6 (-3.3, 5.3) | -5.3 (-7.8, -1.4) | 6.8 (2.5, 9.2) |  |
| **Fibre density at baseline**, g/MJ | 2.3 (1.9, 2.6) | 2.5 (2.2, 2.8) | 2.1 (1.6, 2.4) |  |
| **Fibre density change 0-6m**, g/MJ | 0.1 (-0.2, 0.5) | -0.2 (-0.5, 0.1) | 0.5 (0.1, 1.0) |  |
| **SFA at baseline**, %TEI | 11 (9.5, 13.1) | 9.9 (8.8, 11.5) | 12.6 (10.5, 16.1) |  |
| **SFA change 0-6m**, %TEI | 0.1 (-2.1, 1.8) | 1.8 (0.2, 3.4) | -2.5 (-5.2, -0.4) |  |
| **MUFA at baseline**, %TEI | 12.1 (10.5, 13.7) | 10.9 (9.4, 12.7) | 13.6 (11.4, 15) |  |
| **MUFA change 0-6m**, %TEI | 0.2 (-1.5, 2.0) | 1.8 (0.9, 3.5) | -1.7 (-3.3, 0.2) |  |
| **PUFA at baseline**, %TEI | 6.4 (5.3, 7.8) | 6.0 (5.0, 7.2) | 6.6 (5.1, 7.9) |  |
| **PUFA change 0-6m**, %TEI | 0.1 (-1.4, 1.6) | 0.4 (-1.1, 2.4) | 0.1 (-1.4, 2.1) |  |
| Total fat at baseline, %TEI | 33.8 (30.3, 37.0) | 30.5 (25.8, 34.8) | 36.9 (33.6, 39.8) |  |
| Total fat change 0-6m, %TEI | 0.1 (-3.8, 3.7) | 4.7 (2.0, 8.3) | -4.6 (-8.0, -1.7) |  |
| Total carbohydrate at baseline, %TEI | 45.4 (41.4, 49.9) | 48.1 (44.6, 53.6) | 41.9 (37.1, 44.8) |  |
| Total carbohydrate change 0-6m, %TEI | 0.7 (-3.1, 5.4) | -5.6 (-7.9, -1.8) | 7.3 (2.9, 9.7) |  |
|  |  |  |  |  |
| ***Positive factor loading food group intakes*** |  |  |  |  |
| Fruit (fresh) at baseline, g/d | 165 (96, 246) | 181 (132, 288) | 144 (67, 241) |  |
| *% consumers at baseline* | *93* | *96* | *85* |  |
| Fruit (fresh) change 0-6m, g/d | -17 (-60, 51) | -46 (-147, -22) | 47 (-24, 134) |  |
| *% consumers change 0-6m* | *2* | *0* | *9* |  |
| Low fat milk at baseline, g/d | 182 (118, 253) | 192 (130, 250) | 185 (91, 255) |  |
| *% consumers at baseline* | *92* | *94* | *94* |  |
| Low fat milk change 0-6m, g/d | -13 (-60, 48) | -32 (-104, 28) | 15 (-42, 87) |  |
| *% consumers change 0-6m* | *3* | *-2* | *2* |  |
| Boiled/baked potatoes at baseline, g/d | 44 (0, 83) | 70 (38, 100) | 25 (0, 64) |  |
| *% consumers at baseline* | *72* | *82* | *54* |  |
| Boiled/baked potatoes change 0-6m, g/d | 0 (-35, 46) | -15 (-47, 31) | 16 (0, 53) |  |
| *% consumers change 0-6m* | *5* | *-4* | *25* |  |
| Legumes at baseline, g/d | 18 (0, 48) | 19 (0, 53) | 17 (0, 31) |  |
| *% consumers at baseline* | *69* | *63* | *63* |  |
| Legumes change 0-6m, g/d | 0 (-17, 20) | 0 (-26, 18) | 7 (-1, 40) |  |
| *% consumers change 0-6m* | *2* | *-6* | *18* |  |
| Meat substitutes at baseline, g/d | 0 (0, 0) | 0 (0, 0) | 0 (0, 0) |  |
| *% consumers at baseline* | *2* | *2* | *0* |  |
| Meat substitutes change 0-6m, g/d | 0 (0, 0) | 0 (0, 0) | 0 (0, 0) |  |
| *% consumers change 0-6m* | *0* | *2* | *2* |  |
| **Negative factor loading food group intakes** |  |  |  |  |
| Higher fat processed meats at baseline, g/d | 10 (0, 30) | 0 (0, 23) | 17 (0, 41) |  |
| *% consumers at baseline* | *56* | *49* | *60* |  |
| Higher fat processed meats change 0-6m, g/d | 0 (-15, 12) | 0 (-10, 15) | 0 (-31, 7) |  |
| *% consumers change 0-6m* | *0* | *8* | *-12* |  |
| Butter and animal fats at baseline, g/d | 0 (0, 0) | 0 (0, 0) | 0 (0, 11) |  |
| *% consumers at baseline* | *23* | *14* | *44* |  |
| Butter and animal fats change 0-6m, g/d | 0 (0, 0) | 0 (0, 0) | 0 (-7, 0) |  |
| *% consumers change 0-6m* | *0* | *13* | *-25* |  |
| Red meat at baseline, g/d | 16 (0, 35) | 15 (0, 27) | 26 (0, 56) |  |
| *% consumers at baseline* | *54* | *53* | *65* |  |
| Red meat change 0-6m, g/d | -159 (-241, -91) | -160 (-241, -93) | -154 (-233, -77) |  |
| *% consumers change 0-6m* | *3* | *8* | *-7* |  |
| Low fibre bread at baseline, g/d | 2 (0, 35) | 0 (0, 28) | 20 (0, 34) |  |
| *% consumers at baseline* | *50* | *45* | *63* |  |
| Low fibre bread change 0-6m, g/d | 0 (-23, 0) | 0 (-8, 0) | -5 (-28, 0) |  |
| *% consumers change 0-6m* | *-14* | *-14* | *-23* |  |
| Fried/roast, chips & other potato with added fat at baseline, g/d | 36 (0, 68) | 18 (0, 50) | 39 (0, 75) |  |
| *% consumers at baseline* | *68* | *57* | *71* |  |
| Fried/roast, chips & other potato with added fat change 0-6m, g/d | 0 (-34, 13) | 0 (-14, 13) | -6 (-48, 8) |  |
| *% consumers change 0-6m* | *-6* | *4* | *-19* |  |
| **Age at baseline**, years | 62 (57, 69) | 63 (57, 71) | 61 (56, 65) |  |
| **Time since diagnosis at baseline**, years | 0.5 (0.4, 0.6) | 0.5 (0.4, 0.6) | 0.5 (0.4, 0.6) |  |
| **Male**, n (%) | 163 (67%) | 31 (63%) | 37 (77%) |  |
| **Under-reporting at baseline**, n (%) | 135 (56%) | 24 (49%) | 20 (42%) |  |
| **IMD at baseline** | 12.6 (6.4, 18.9) | 11.9 (6.4, 16.7) | 13.6 (5.9, 20.9) |  |
| **HbA1c at baseline**, mmol/mol | 47 (43, 54) | 48 (44, 54) | 45 (40, 55) |  |
| **HbA1c at baseline**, % | 6.5 (6.1, 7.1) | 6.5 (6.2, 7.1) | 6.3 (5.9, 7.2) |  |
| **HbA1c change 0-6m**, mmol/mol | -2.2 (-5.5, 3.3) | -1.1 (-4.4, 2.2) | -2.2 (-6.6, 4.9) |  |
| **HbA1c change 0-6m**, % | -0.2 (-0.5, 0.3) | -0.1 (-0.4, 0.2) | -0.2 (-0.6, 0.5) |  |
| **Weight at baseline**, kg | 86.6 (77.1, 94) | 86.5 (78.9, 92.6) | 82.7 (75.1, 93.6) |  |
| **Weight change 0-6m**, kg | -2.1 (-3.9, -0.1) | -1.9 (-3.3, 0.0) | -2.9 (-5.1, -0.9) |  |
| **BMI at baseline**, kg/m^2^ | 29.5 (27.3, 32.7) | 29 (26.8, 32.9) | 29.3 (27.2, 32.8) |  |
| **BMI change 0-6m**, kg/m^2^ | -0.7 (-1.4, 0) | -0.6 (-1.2, 0.0) | -1.1 (-2.1, -0.3) |  |
| **Total physical activity at baseline**, counts/min | 291 (226, 363) | 286 (210, 348) | 308 (256, 390) |  |
| **Total physical activity change 0-6m**, counts/min | 16 (-44, 91) | 18 (-42, 66) | 37 (-31, 115) |  |
| **MVPA at baseline**, mins/day | 21 (13, 36) | 19 (13, 37) | 25 (16, 39) |  |
| **MVPA change 0-6m**, mins/day | 3 (-6, 18) | 0 (-8, 12) | 7 (0, 24) |  |
| **Metformin**, n (%) | 85 (35%) | 11 (22%) | 17 (35%) |  |
| **Metformin**, %max dose | 0 (0, 50) | 0 (0, 0) | 0 (0, 50) |  |
| **Sulphonylurea**, n (%) | 22 (9%) | 5 (10%) | 6 (13%) |  |
| **Sulphonylurea**, %max dose | 0 (0, 0) | 0 (0, 0) | 0 (0, 0) |  |
| **Glitazone**, n (%) | 2 (1%) | 0 (0%) | 0 (0%) |  |
| **Glitazone**, %max dose | 0 (0, 0) | 0 (0, 0) | 0 (0, 0) |  |

*TEI – total energy intake; SFA – saturated fat; MUFA – monounsaturated fat; PUFA – polyunsaturated fat; IMD – index of multiple deprivation; MVPA – moderate-vigorous physical activity.*

**Table S6**: Descriptive dietary and sample characteristics by quintile of ‘obesogenic’ dietary pattern score change during 0-6m. Participants in quintile 1 made the greatest change towards less energy-dense, lower-fat, higher-fibre intakes during 0-6 months (mean score change: -1.52, SD 0.44). Participants in quintile 5 made the least dietary change in this direction (mean change: 1.08, SD 0.44; suggesting average participant dietary intakes in this quintile became more energy-dense, higher in fat and lower in fibre compared to baseline intakes). All other data presented as n (%) or median (Q1, Q3). Highest loading food group intakes in median (Q1, Q3) g/day are presented to indicate the average amounts (and change in amounts) of foods consumed. Percentage of consumers (and change in consumers) of these food groups indicate the number of (and change in number of) individuals consuming any amount of these foods. Median ‘Meat substitutes’ intake is zero, for example, but should be interpreted in context of percentage of consumers being very low (2% of whole sample).

| **Highest and lowest quintile sample characteristics (models 1a-3) of 0-6m ‘obesogenic’ dietary pattern score change** | | | |
| --- | --- | --- | --- |
|  | **Whole sample** | **Quintile 1** | **Quintile 5** |
| **N** | 242 | 49 | 48 |
| **Obesogenic dietary pattern score at baseline**, SD | -0.05±0.97 | 0.71±0.84 | -0.71±0.91 |
| **Obesogenic dietary pattern score change 0-6m**, SD | -0.24±0.94 | -1.52±0.44 | 1.08±0.44 |
| **TEI at baseline**, kJ | 7347 (6220, 8619) | 8175 (6910, 9574) | 7466 (6525, 8591) |
| **TEI change 0-6m**, kJ | -731 (-1647, -6) | -1072 (-2036, -321) | -272 (-1368, 538) |
| **DED at baseline**, kJ/g | 6.3 (5.6, 7.1) | 7.2 (6.4, 7.8) | 5.8 (5.3, 6.4) |
| **DED change 0-6m**, kJ/g | -0.3 (-1.0, 0.5) | -1.5 (-1.8, -0.9) | 0.8 (0.3, 1.3) |
| **Fibre density at baseline**, g/MJ | 2.3 (1.9, 2.6) | 2.0 (1.7, 2.4) | 2.4 (2.1, 2.7) |
| **Fibre density change 0-6m**, g/MJ | 0.1 (-0.2, 0.5) | 0.7 (0.4, 1.0) | -0.3 (-0.6, 0.0) |
| **Total fat at baseline**, %TEI | 33.8 (30.3, 37.0) | 35.8 (34.3, 39.0) | 33.4 (28.4, 35.8) |
| **Total fat change 0-6m**, %TEI | 0.1 (-3.8, 3.7) | -3.8 (-6.6, 0.1) | 2.6 (0.1, 7.0) |
| Total carbohydrate at baseline, %TEI | 45.4 (41.4, 49.9) | 42.8 (38.5, 45.8) | 46.4 (43.0, 50.8) |
| Total carbohydrate change 0-6m, %TEI | 0.7 (-3.1, 5.4) | 5.5 (0.2, 9.3) | -2.5 (-6.8, 1.5) |
| SFA at baseline, %TEI | 11.0 (9.5, 13.1) | 11.9 (10.4, 15.5) | 10.8 (9.4, 12.2) |
| SFA change 0-6m, %TEI | 0.1 (-2.1, 1.8) | -1.6 (-3.7, 0.2) | 0.3 (-1.2, 3.0) |
| ***Positive factor loading food group intakes*** |  |  |  |
| Low fibre bread at baseline, g/d | 2 (0, 35) | 18 (0, 33) | 0 (0, 21) |
| *% consumers at baseline* | *50* | *57* | *33* |
| Low fibre bread change 0-6m, g/d | 0 (-23, 0) | -5 (-30, 0) | 0 (0, 1) |
| *% consumers change 0-6m* | *-14* | *-24* | *2* |
| Processed meat at baseline, g/d | 27 (9, 51) | 28 (11, 51) | 23 (3, 54) |
| *% consumers at baseline* | *82* | *76* | *75* |
| Processed meat change 0-6m, g/d | -1 (-23, 15) | 4 (-16, 15) | 0 (-28, 31) |
| *% consumers change 0-6m* | *1* | *10* | *0* |
| Coated chicken & fish at baseline, g/d | 0 (0, 0) | 0 (0, 0) | 0 (0, 0) |
| *% consumers at baseline* | *21* | *22* | *17* |
| Coated chicken & fish change 0-6m, g/d | 0 (0, 0) | 0 (0, 0) | 0 (0, 0) |
| *% consumers change 0-6m* | *-3* | *-2* | *6* |
| Fried/roast potatoes & chips at baseline, g/d | 25 (0, 49) | 33 (0, 50) | 25 (0, 47) |
| *% consumers at baseline* | *60* | *65* | *52* |
| Fried/roast potatoes & chips change 0-6m, g/d | 0 (-26, 13) | 0 (-30, 13) | 0 (-12, 15) |
| *% consumers change 0-6m* | *-7* | *-10* | *0* |
| Biscuits & cakes at baseline, g/d | 14 (0, 33) | 19 (4, 37) | 8 (0, 29) |
| *% consumers at baseline* | *71* | *78* | *58* |
| Biscuits & cakes change 0-6m, g/d | 0 (-17, 7) | -5 (-21, 1) | 0 (-9, 18) |
| *% consumers change 0-6m* | *-9* | *-13* | *7* |
| ***Negative factor loading food group intakes*** |  |  |  |
| Fruit (fresh) at baseline, g/d | 165 (96, 246) | 122 (63, 229) | 185 (103, 289) |
| *% consumers at baseline* | *93* | *94* | *96* |
| Fruit (fresh) change 0-6m, g/d | -17 (-60, 51) | 69 (0, 138) | -56 (-170, -19) |
| *% consumers change 0-6m* | *2* | *2* | *-2* |
| Vegetables (Raw/boiled/grilled) at baseline, g/d | 120 (88, 173) | 104 (83, 147) | 138 (89, 211) |
| *% consumers at baseline* | *99* | *100* | *98* |
| Vegetables (Raw/boiled/grilled) change 0-6m, g/d | 5 (-42, 56) | 57 (0, 101) | -34 (-60, 4) |
| *% consumers change 0-6m* | *0* | *-2* | *0* |
| Yoghurts at baseline, g/d | 0 (0, 61) | 0 (0, 31) | 31 (0, 91) |
| *% consumers at baseline* | *46* | *39* | *65* |
| Yoghurts change 0-6m, g/d | 0 (-13, 15) | 0 (0, 20) | 0 (-31, 3) |
| *% consumers change 0-6m* | *-1* | *0* | *-11* |
| Boiled/baked potatoes at baseline, g/d | 55 (30, 92) | 44 (0, 75) | 66 (39, 101) |
| *% consumers at baseline* | *81* | *67* | *81* |
| Boiled/baked potatoes change 0-6m, g/d | 0 (-38, 45) | 21 (0, 48) | -13 (-49, 35) |
| *% consumers change 0-6m* | *4* | *21* | *0* |
| Meat substitutes at baseline, g/d | 0 (0, 0) | 0 (0, 0) | 0 (0, 0) |
| *% consumers at baseline* | *2* | *2* | *2* |
| Meat substitutes change 0-6m, g/d | 0 (0, 0) | 0 (0, 0) | 0 (0, 0) |
| *% consumers change 0-6m* | *0* | *-2* | *2* |
| **Age at baseline**, years | 62 (57, 69) | 61 (55, 66) | 61 (53, 68) |
| **Time since diagnosis at baseline**, years | 0.5 (0.4, 0.6) | 0.5 (0.4, 0.6) | 0.5 (0.4, 0.6) |
| **Male**, n (%) | 163 (67%) | 36 (73%) | 31 (65%) |
| **Under-reporting at baseline**, n (%) | 135 (56%) | 21 (43%) | 25 (52%) |
| **IMD at baseline** | 12.6 (6.4, 18.9) | 12.8 (6.3, 20.5) | 8.3 (5.4, 17.6) |
| **HbA1c at baseline**, mmol/mol | 47 (43, 54) | 47 (43, 52) | 49 (44, 57) |
| **HbA1c at baseline**, % | 6.5 (6.1, 7.1) | 6.4 (6.1, 6.9) | 6.6 (6.2, 7.4) |
| **HbA1c change 0-6m**, mmol/mol | -2.2 (-5.5, 3.3) | -2.2 (-6.6, 3.3) | -1.1 (-6.6, 2.7) |
| **HbA1c change 0-6m**, % | -0.2 (-0.5, 0.3) | -0.2 (-0.6, 0.3) | -0.1 (-0.6, 0.2) |
| **Weight at baseline**, kg | 86.6 (77.1, 94) | 88.1 (75.5, 95) | 85.8 (78.2, 93.6) |
| **Weight change 0-6m**, kg | -2.1 (-3.9, -0.1) | -2.4 (-4.5, -0.4) | -1.6 (-3.1, 1.1) |
| **BMI at baseline**, kg/m^2^ | 29.5 (27.3, 32.7) | 29.4 (26.5, 32.3) | 29.2 (26.7, 33) |
| **BMI change 0-6m**, kg/m^2^ | -0.7 (-1.4, 0.0) | -0.8 (-1.5, -0.1) | -0.5 (-1.1, 0.4) |
| **Total physical activity at baseline**, counts/min | 291 (226, 363) | 302 (244, 356) | 294 (241, 352) |
| **Total physical activity change 0-6m**, counts/min | 16 (-44, 91) | 35 (-2, 117) | 4 (-66, 66) |
| **MVPA at baseline**, mins/day | 21 (13, 36) | 23 (14, 37) | 25 (16, 42) |
| **MVPA change 0-6m**, mins/day | 3 (-6, 18) | 7 (-3, 25) | -2 (-9, 10) |
| **Metformin**, n (%) | 85 (35%) | 20 (41%) | 15 (31%) |
| **Metformin**, %max dose | 0 (0, 50) | 0 (0, 50) | 0 (0, 50) |
| **Sulphonylurea**, n (%) | 22 (9%) | 7 (14%) | 5 (10%) |
| **Sulphonylurea**, %max dose | 0 (0, 0) | 0 (0, 0) | 0 (0, 0) |
| **Glitazone,** n (%) | 2 (1%) | 0 (0%) | 0 (0%) |
| **Glitazone**, %max dose | 0 (0, 0) | 0 (0, 0) | 0 (0, 0) |

*TEI – total energy intake; DED – dietary energy-density; SFA – saturated fat; IMD – index of multiple deprivation; MVPA – moderate-vigorous physical activity.*

**Table S7**: Relation between change in HbA1c (mmol/mol) and change in dietary pattern scores during study periods. β is the change in end-of-period HbA1c associated with a 1-SD positive increase in dietary pattern score during period.

|  |  |  |  | **Carb/fat balance pattern** | | |  | **Obesogenic pattern** | | |
| --- | --- | --- | --- | --- | --- | --- | --- | --- | --- | --- |
| **Timepoint** | **Model** | **n** |  | **β** | **95%CI** | **p** |  | **β** | **95%CI** | **p** |
| 0-6 months | *1* | 280 |  | -2.16 | -3.42, -0.91 | **0.001** |  | 1.04 | 0.01, 2.07 | **0.047** |
|  | *1a* | 242 |  | -2.27 | -3.69, -0.84 | **0.002** |  | 1.17 | 0.02, 2.33 | **0.046** |
|  | *2* | 242 |  | -2.21 | -3.65, -0.78 | **0.003** |  | 1.06 | -0.10, 2.23 | 0.074 |
|  | *3* | 242 |  | -1.54 | -2.96, -0.13 | **0.033** |  | 0.63 | -0.52, 1.78 | 0.283 |
| 6-12 months | *1* | 229 |  | -0.42 | -1.47, 0.62 | 0.426 |  | 0.26 | -0.66, 1.17 | 0.580 |
|  | *1a* | 194 |  | -0.25 | -1.38, 0.88 | 0.663 |  | 0.09 | -0.88, 1.06 | 0.854 |
|  | *2* | 194 |  | -0.46 | -1.61, 0.68 | 0.427 |  | 0.17 | -0.81, 1.16 | 0.727 |
|  | *3* | 194 |  | -0.61 | -1.83, 0.60 | 0.321 |  | 0.28 | -0.76, 1.33 | 0.591 |
| 0-12 months | *1* | 256 |  | -1.31 | -2.34, -0.27 | **0.014** |  | 0.80 | -0.09, 1.70 | 0.079 |
|  | *1a* | 214 |  | -0.68 | -1.82, 0.46 | 0.240 |  | 0.48 | -0.50, 1.46 | 0.335 |
|  | *2* | 214 |  | -0.86 | -2.00, 0.28 | 0.139 |  | 0.60 | -0.40, 1.59 | 0.237 |
|  | *3* | 214 |  | -0.78 | -2.02, 0.45 | 0.212 |  | 0.51 | -0.53, 1.55 | 0.335 |

*1 – linear regression adjusted for start-of-period HbA1c and dietary pattern score in those with complete HbA1c and dietary pattern data during period.
1a – model 1 in sample restricted to those with complete covariate data.
2 – model 1a adjusted for age, sex, baseline under-reporting, change in OHA medications (metformin, sulphonylurea and glitazones separately in percentages of maximum dose) and change in total physical activity during period.
3 – model 2 adjusted for change in TEI and bodyweight during period (potential mediators between dietary pattern and HbA1c change).*

**Table S8**: Relation between change in HbA1c (mmol/mol) and change in bodyweight during study periods. β is the change in end-of-period HbA1c associated with a 1kg increase in bodyweight during period.

| **Timepoint** | **Model** | **n** | **β** | **95%CI** | **p** |
| --- | --- | --- | --- | --- | --- |
| 0-6 months | *1* | 564 | 0.85 | 0.68, 1.03 | **<0.001** |
|  | *1a* | 242 | 0.72 | 0.47, 0.97 | **<0.001** |
|  | *2* | 242 | 0.70 | 0.45, 0.95 | **<0.001** |
| 6-12 months | *1* | 549 | 0.17 | -0.04, 0.37 | 0.121 |
|  | *1a* | 194 | 0.11 | -0.23, 0.45 | 0.524 |
|  | *2* | 194 | 0.07 | -0.27, 0.41 | 0.670 |
| 0-12 months | *1* | 559 | 0.45 | 0.31, 0.60 | **<0.001** |
|  | *1a* | 214 | 0.41 | 0.22, 0.60 | **<0.001** |
|  | *2* | 214 | 0.37 | 0.17, 0.56 | **<0.001** |

*1 – linear regression adjusted for start-of-period bodyweight and HbA1c in those with complete bodyweight and HbA1c data during period.
1a – model 1 in sample restricted to those with complete covariate and diet pattern data.
2 – model 1a adjusted for age, sex and change in total physical activity during period.*

**Table S9**: Relation between change in bodyweight (kg) and change in dietary pattern score during study periods. β is the change in end-of-period bodyweight associated with a 1-SD positive increase in dietary pattern score during period.

|  |  |  |  | **Carb/fat balance pattern** | | |  | **Obesogenic pattern** | | |
| --- | --- | --- | --- | --- | --- | --- | --- | --- | --- | --- |
| **Timepoint** | **Model** | **n** |  | **β** | **95%CI** | **p** |  | **β** | **95%CI** | **p** |
| 0-6 months | *1* | 280 |  | -1.06 | -1.66, -0.47 | **<0.001** |  | 0.62 | 0.14, 1.11 | **0.013** |
|  | *1a* | 242 |  | -1.22 | -1.89, -0.55 | **<0.001** |  | 0.81 | 0.26, 1.35 | **0.004** |
|  | *2* | 242 |  | -1.22 | -1.89, -0.55 | **<0.001** |  | 0.77 | 0.23, 1.31 | **0.006** |
|  | *3* | 242 |  | -1.18 | -1.86, -0.51 | **<0.001** |  | 0.73 | 0.18, 1.29 | **0.010** |
| 6-12 months | *1* | 229 |  | -0.42 | -0.88, 0.03 | 0.070 |  | 0.20 | -0.20, 0.60 | 0.324 |
|  | *1a* | 194 |  | -0.28 | -0.75, 0.20 | 0.248 |  | 0.12 | -0.28, 0.53 | 0.547 |
|  | *2* | 194 |  | -0.34 | -0.82, 0.13 | 0.158 |  | 0.20 | -0.21, 0.62 | 0.341 |
|  | *3* | 194 |  | -0.21 | -0.71, 0.29 | 0.417 |  | 0.06 | -0.38, 0.50 | 0.782 |
| 0-12 months | *1* | 255 |  | -1.14 | -1.85, -0.42 | **0.002** |  | 0.68 | 0.06, 1.30 | **0.032** |
|  | *1a* | 214 |  | -0.87 | -1.65, -0.10 | **0.028** |  | 0.50 | -0.17, 1.18 | 0.140 |
|  | *2* | 214 |  | -1.06 | -1.83, -0.30 | **0.007** |  | 0.71 | 0.04, 1.38 | **0.039** |
|  | *3* | 214 |  | -0.98 | -1.83, -0.14 | **0.023** |  | 0.60 | -0.11, 1.32 | 0.098 |

*1 – linear regression adjusted for start-of-period bodyweight and dietary pattern score in those with complete bodyweight, HbA1c and dietary pattern data during period.
1a – model 1 in sample restricted to those with complete covariate data.
2 – model 1a adjusted for age, sex, baseline under-reporting and change in total physical activity during period.
3 – model 2 adjusted for change in TEI during period (potential mediator between dietary pattern and bodyweight change).*

**Table S10**: Relation between change in HbA1c (mmol/mol) and change in dietary pattern scores during study periods, with additional adjustment for trial arm in models 2-3. β is the change in end-of-period HbA1c associated with a 1-SD positive increase in dietary pattern score during period.

|  |  |  |  | **Carb/fat balance pattern** | | |  | **Obesogenic pattern** | | |
| --- | --- | --- | --- | --- | --- | --- | --- | --- | --- | --- |
| **Timepoint** | **Model** | **n** |  | **β** | **95%CI** | **p** |  | **β** | **95%CI** | **p** |
| 0-6 months | *1* | 280 |  | -2.16 | -3.42, -0.91 | **<0.001** |  | 1.04 | 0.01, 2.07 | **0.047** |
|  | *1a* | 242 |  | -2.27 | -3.69, -0.84 | **0.002** |  | 1.17 | 0.02, 2.33 | **0.046** |
|  | *2* | 242 |  | -1.88 | -3.32, -0.44 | **0.011** |  | 0.80 | -0.37, 1.96 | 0.179 |
|  | *3* | 242 |  | -1.38 | -2.80, 0.05 | 0.058 |  | 0.48 | -0.67, 1.64 | 0.408 |
| 6-12 months | *1* | 229 |  | -0.42 | -1.47, 0.62 | 0.426 |  | 0.26 | -0.66, 1.17 | 0.580 |
|  | *1a* | 194 |  | -0.25 | -1.38, 0.88 | 0.663 |  | 0.09 | -0.88, 1.06 | 0.854 |
|  | *2* | 194 |  | -0.38 | -1.56, 0.80 | 0.530 |  | 0.11 | -0.89, 1.11 | 0.831 |
|  | *3* | 194 |  | -0.54 | -1.78, 0.71 | 0.397 |  | 0.23 | -0.83, 1.29 | 0.672 |
| 0-12 months | *1* | 256 |  | -1.31 | -2.34, -0.27 | **0.014** |  | 0.80 | -0.09, 1.70 | 0.079 |
|  | *1a* | 214 |  | -0.68 | -1.82, 0.46 | 0.240 |  | 0.48 | -0.50, 1.46 | 0.335 |
|  | *2* | 214 |  | -0.82 | -1.97, 0.33 | 0.159 |  | 0.58 | -0.42, 1.58 | 0.254 |
|  | *3* | 214 |  | -0.76 | -2.01, 0.49 | 0.231 |  | 0.50 | -0.56, 1.55 | 0.353 |

*1 – linear regression adjusted for start-of-period HbA1c and dietary pattern score in those with complete HbA1c and dietary pattern data during period.
1a – model 1 in sample restricted to those with complete covariate data.
2 – model 1a adjusted for trial arm, age, sex, baseline under-reporting, change in OHA medications (metformin, sulphonylurea and glitazones separately in percentages of maximum dose) and change in total physical activity during period.
3 – model 2 adjusted for change in TEI and bodyweight during period (potential mediators between dietary pattern and HbA1c change).*

**Fig. S1*:*** Dietary pattern factor loading diagrams. Positive food group factor loadings increase dietary pattern scores, whilst negative loadings decrease dietary pattern scores. Highest and lowest loading food groups have the greatest impact on dietary pattern scores when changing intakes. *a*: ‘Carb/fat balance’ dietary pattern; *b*: ‘Obesogenic’ dietary pattern.


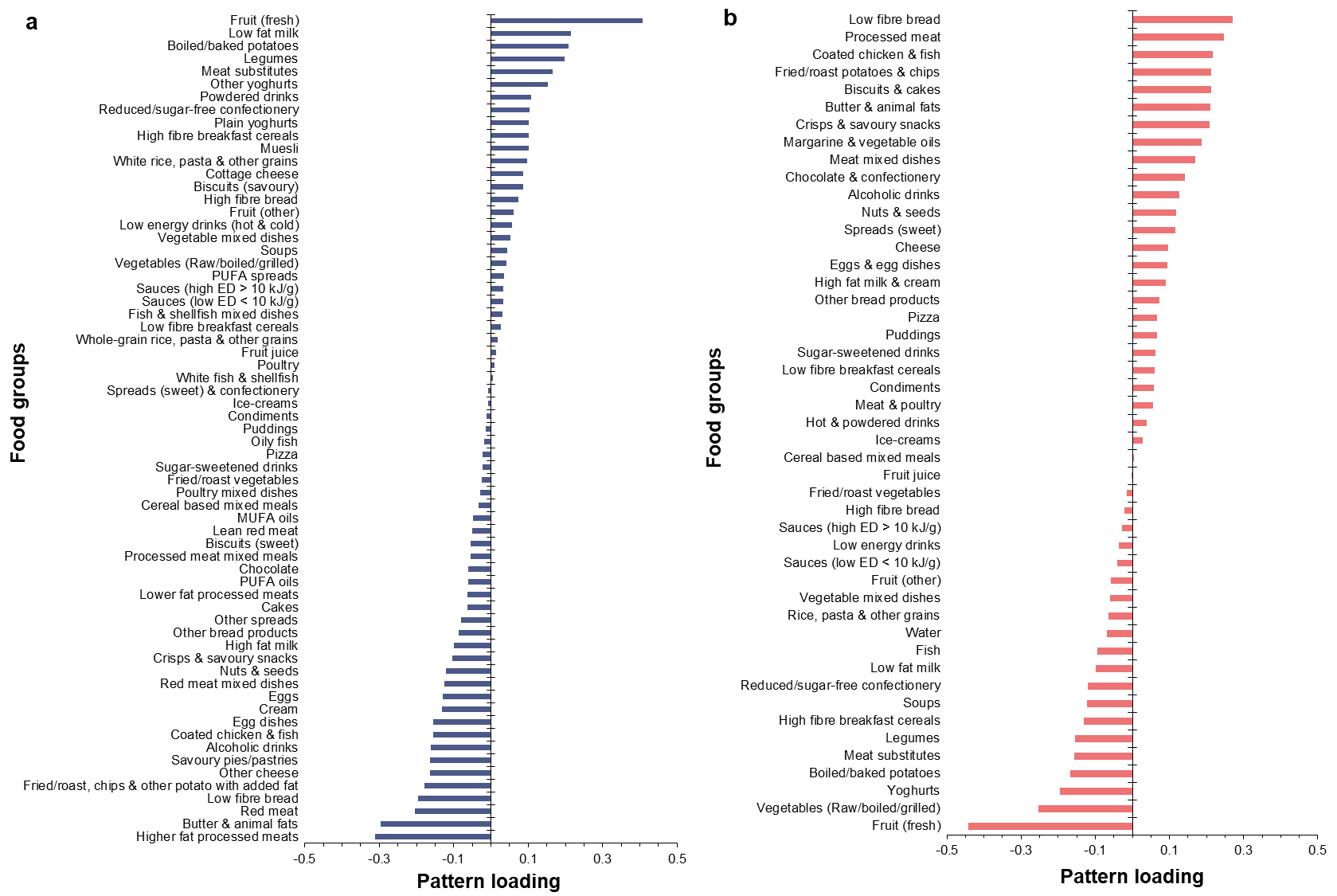


**Fig. S2*:*** Associations between 1-SD increases in dietary pattern scores during 6-12 or 0-12 month periods and start-of-period-adjusted HbA1c at 12-months from multivariable linear regression. *Model 1a* presents *Model 1* start-of-period dietary pattern score adjusted associations in those with complete covariate data. *Model 2* presents associations adjusted for potential confounders: age, sex, baseline under-reporting status, and change in total physical activity and percentage of maximum metformin, sulphonylurea and glitazone doses. *Model 3* presents Model 2 associations adjusted for potential mediators: change in bodyweight and energy intake. *a*: Associations between a 1-SD increase in ‘carb/fat balance’ dietary pattern score and end-of-period change in HbA1c; b: Associations between 1-SD increases in ‘obesogenic’ dietary pattern score and end-of-period change in HbA1c.


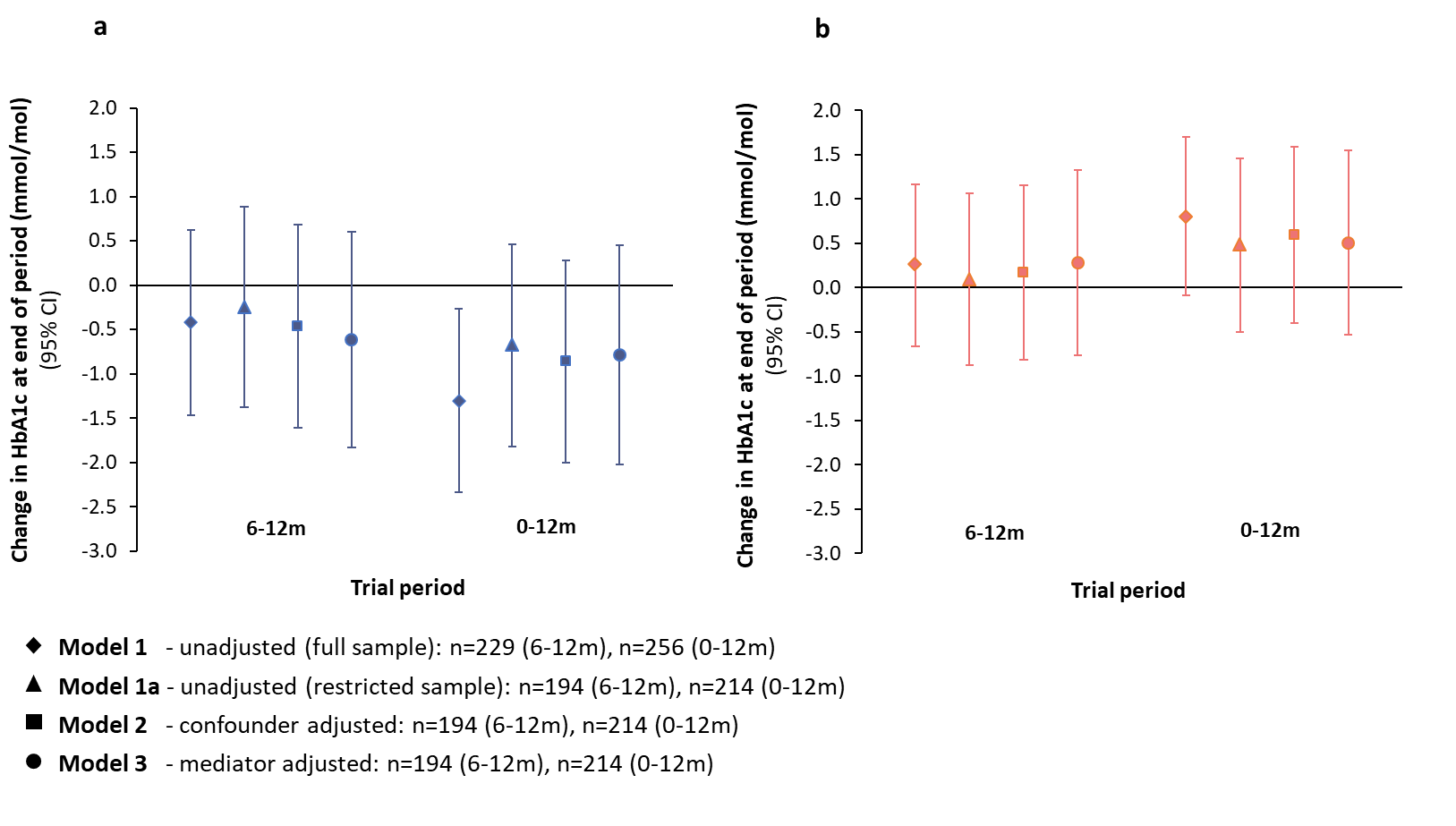


**Fig. S3*:*** Fitted lines by sex from simple linear regression analyses of end-of-period HbA1c on ‘carb/fat balance’ and ‘obesogenic’ dietary pattern (DP) change, to investigate potential interactions between DP change and sex. p_int_ – p-value for DP*sex interaction term calculated within model 2 (confounding-adjusted regression models). *a*: 6-month HbA1c on 0-6m ‘carb/fat balance’ DP change (p_int_=0.98); *b*: 12-month HbA1c on 6-12m ‘carb/fat balance’ DP change (p_int_=0.14); *c*: 12-month HbA1c on 0-12m ‘carb/fat balance’ DP change (p_int_=0.12); *d*: 6-month HbA1c on 0-6m ‘obesogenic’ DP change (p_int_=0.78); *e*: 12-month HbA1c on 6-12m ‘obesogenic’ DP change (p_int_=0.23); *f*: 12-month HbA1c on 0-12m ‘obesogenic’ DP change (p_int_=0.10).


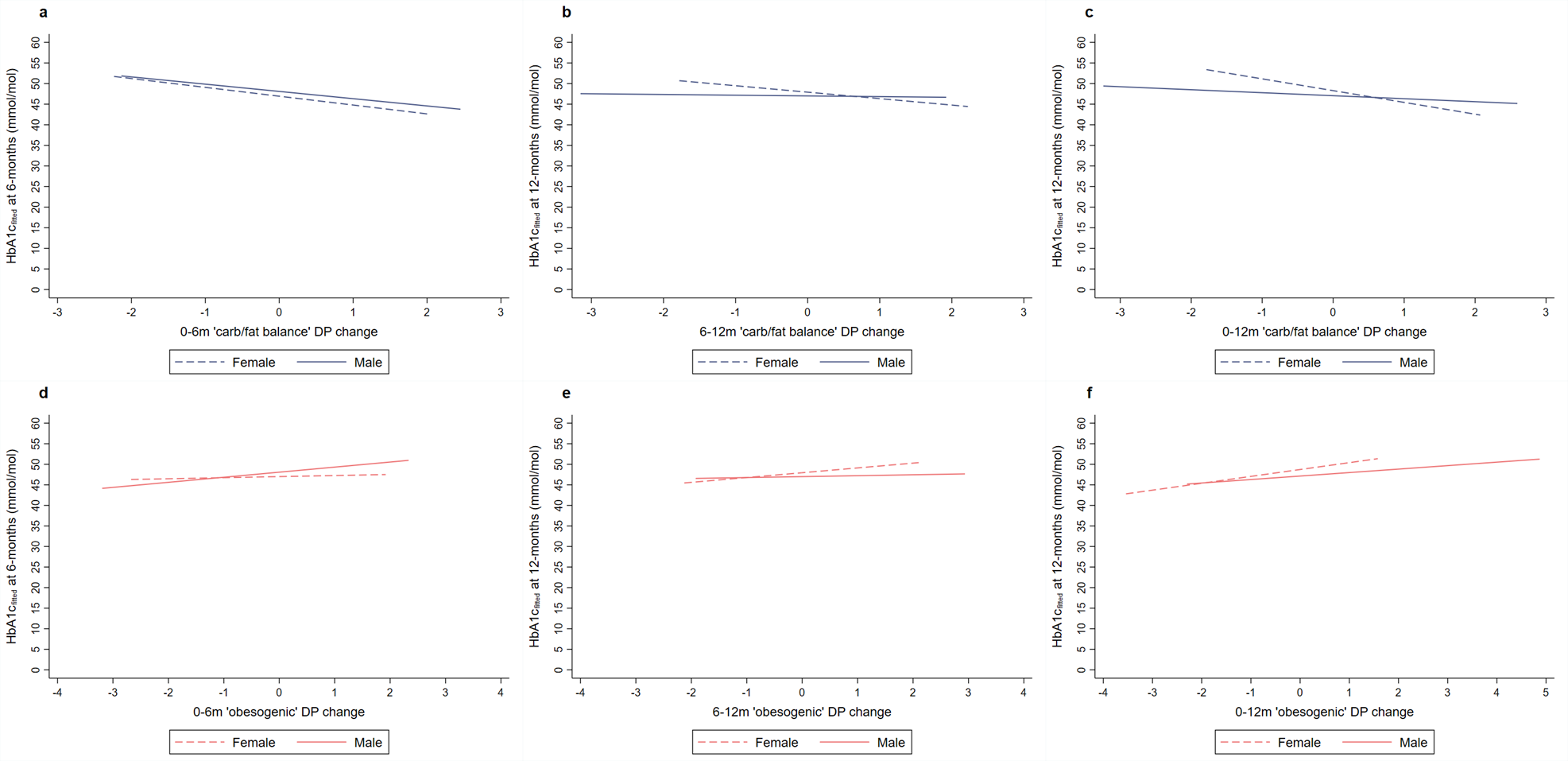


**Fig. S4**: Linear trend assessment. Estimated marginal mean (95%CI) plots for HbA1c regressed on ‘carb/fat balance’ and ‘obesogenic’ dietary pattern (DP) score change quintiles for confounding-adjusted 0-6m, 6-12m and 0-12m period models (model 2). p_trend_ – model 2 linear test for trend performed using likelihood ratio test; modelling DP score change as a nested categorical (quintile) variable did not improve model fit compared to DP score change modelled as a continuous variable. Indications of a non-linear trend in panel *e* should be interpreted alongside the lack of evidence for non-linear trend in all other panels. *a*: 6-month HbA1c on 0-6m ‘carb/fat balance’ DP change quintiles (p_trend_=0.27); *b*: 12-month HbA1c on 6-12m ‘carb/fat balance’ DP change quintiles (p_trend_=0.40); *c*: 12-month HbA1c on 0-12m ‘carb/fat balance’ DP change quintiles (p_trend_=0.31); *d*: 6-month HbA1c on 0-6m ‘obesogenic’ DP change quintiles (p_trend_=0.41); *e*: 12-month HbA1c on 6-12m ‘obesogenic’ DP change quintiles (p_trend_=0.02); *f*: 12-month HbA1c on 0-12m ‘obesogenic’ DP change quintiles (p_trend_=0.18).

##
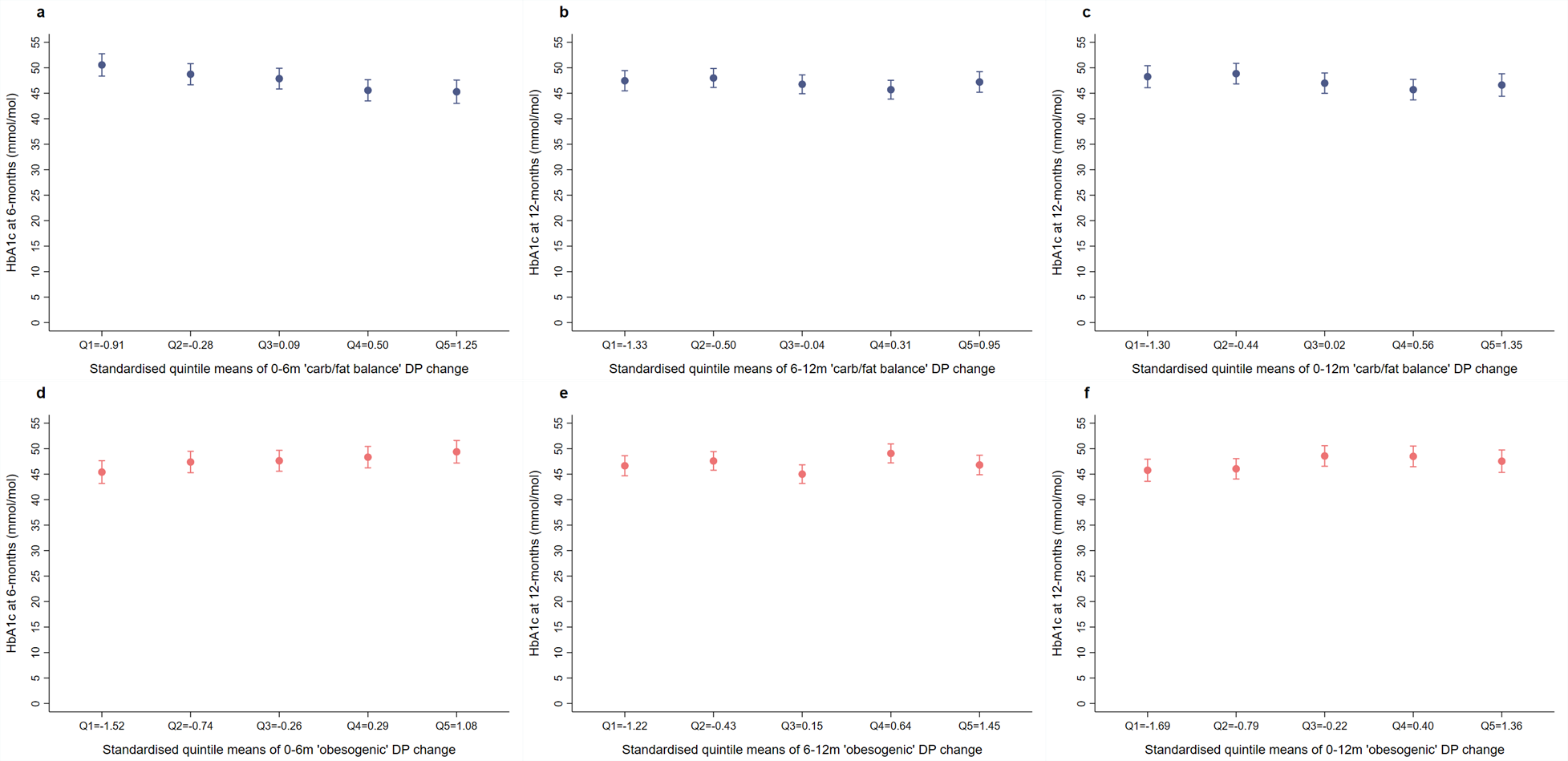

Supplement: Supplementary file 1 — Additional file 1: Supplementary information S1. Further details on dietary misreporting calculation. Supplementary information S2. Further details on sensitivity analyses. Supplementary information S3. Code used for employing reduced-rank regression in Stata (via SAS). Table S1. Food groups and their contents for the ‘obesogenic’ and ‘carb/fat balance’ dietary patterns. Table S2. Characteristics of all Early-ACTID participants compared with participants with complete covariate data for adjusted secondary analyses (periods 6-12m and 0-12m). Table S3. Explained nutrient variation and correlations for ‘carb/fat balance’ dietary patterns derived at 0, 6 and 12 months. Table S4. Explained nutrient variation and nutrient correlations for energy-dense, higher-fat, lower-fibre ‘obesogenic’ dietary patterns derived at 0, 6 and 12 months. Table S5. Descriptive dietary and sample characteristics in extreme quintiles of ‘carb/fat balance’ dietary pattern score change during 0-6m. Table S6. Descriptive dietary and sample characteristics in extreme quintiles of ‘obesogenic’ dietary pattern score change during 0-6m. Table S7. Relation between change in HbA1c and change in dietary pattern scores during study periods. Table S8. Relation between change in HbA1c and change in bodyweight during study periods. Table S9. Relation between change in bodyweight and change in dietary pattern score during study periods. Table S10. Relation between change in HbA1c and change in dietary pattern scores during study periods, with additional adjustment for trial arm in models 2-3. Fig. S1. Dietary pattern factor loading diagrams. Fig. S2. Associations between 1-SD increases in dietary pattern scores during 6-12 or 0-12 month periods and start-of-period-adjusted HbA1c at 12 months from multivariable linear regression. Fig. S3. Fitted lines by sex from simple linear regression analyses of end-of-period HbA1c on ‘carb/fat balance’ and ‘obesogenic’ dietary pattern change, to investigate potential int [file 12916_2022_2358_MOESM1_ESM.docx]
